# Nucleosomal DNA binding drives the recognition of H3K36-methylated nucleosomes by the PSIP1-PWWP domain

**DOI:** 10.1186/1756-8935-6-12

**Published:** 2013-05-08

**Authors:** Rick van Nuland, Frederik MA van Schaik, Marieke Simonis, Sebastiaan van Heesch, Edwin Cuppen, Rolf Boelens, HT Marc Timmers, Hugo van Ingen

**Affiliations:** 1 Molecular Cancer Research, Division of Biomedical Genetics, University Medical Center Utrecht, Universiteitsweg 100, Utrecht, CG, 3584, The Netherlands; 2 Genome Biology Group, Hubrecht Institute, Uppsalalaan 8, Utrecht, CT, 3584, The Netherlands; 3 NMR Spectroscopy Research Group, Bijvoet Center for Biomolecular Research, Utrecht University Utrecht, Padualaan 8, Utrecht, CH, 3854, The Netherlands

**Keywords:** Histone-methylation, H3K36me, Nucleosome, Structure, NMR, Affinity, Specificity, PSIP1, PWWP

## Abstract

**Background:**

Recognition of histone modifications by specialized protein domains is a key step in the regulation of DNA-mediated processes like gene transcription. The structural basis of these interactions is usually studied using histone peptide models, neglecting the nucleosomal context. Here, we provide the structural and thermodynamic basis for the recognition of H3K36-methylated (H3K36me) nucleosomes by the PSIP1-PWWP domain, based on extensive mutational analysis, advanced nuclear magnetic resonance (NMR), and computational approaches.

**Results:**

The PSIP1-PWWP domain binds H3K36me3 peptide and DNA with low affinity, through distinct, adjacent binding surfaces. PWWP binding to H3K36me nucleosomes is enhanced approximately 10,000-fold compared to a methylated peptide. Based on mutational analyses and NMR data, we derive a structure of the complex showing that the PWWP domain is bound to H3K36me nucleosomes through simultaneous interactions with both methylated histone tail and nucleosomal DNA.

**Conclusion:**

Concerted binding to the methylated histone tail and nucleosomal DNA underlies the high- affinity, specific recognition of H3K36me nucleosomes by the PSIP1-PWWP domain. We propose that this bipartite binding mechanism is a distinctive and general property in the recognition of histone modifications close to the nucleosome core.

## Background

Chemical modifications of nucleosomes, the complex of DNA and histone proteins that packages the eukaryotic genome, are key in the regulation of transcription, maintenance of genomic integrity, chromosome condensation and segregation [1]. Modifications such as acetylation or methylation of lysine residues of histone proteins can serve to recruit effector proteins to specific genomic sites [2]. Methylation of lysine-36 in histone H3 (H3K36me) is conserved from yeast to human and is predominantly associated with actively transcribed chromatin [3]. H3K36me has been implicated in diverse processes including splicing, DNA repair, repression of cryptic transcription and histone exchange [4]. Recently, PWWP domains have been identified as H3K36me recognition domains, based on histone peptide interaction studies [5]. PWWP domains feature an aromatic cage, as in other royal Tudor family proteins [6] that bind the methylated lysine side chain via cation-π interactions [7]. Interestingly, interaction studies have shown that PWWP domains bind methylated H3K36 histone tail peptides with very low affinity [8,9], in stark contrast with the high affinity recognition of tri-methylated lysine-4 of H3 (H3K4me3) by PHD fingers [10,11].

Here, we address the structural basis for H3K36me recognition by PWWP domains in the nucleosomal context. Unlike other methylated lysines in the unstructured N-terminal tail of histone H3, K36 is located at the point where the H3 tail protrudes from the nucleosome core [12]. Since PWWP domains were previously also implicated in DNA-binding [13,14], we hypothesized that the close proximity of the nucleosomal DNA may critically contribute to binding affinity and/or specificity of PWWP domains for H3K36me. We concentrate on the PWWP domain containing protein PSIP1, as its association with naked and chromatinized DNA has been the focus of previous studies [15,16] and it was recently identified as a H3K36me-interacting protein using synthetic peptides [17]. PSIP1 (LEDGF/p75) was first isolated as an transcriptional co-activator [18] and tethers the HIV integrase to active host chromatin dependent on its PWWP domain [19,20]. PSIP1 is an essential subunit of the MLL complex in MLL oncogenic transformations via *HOX* gene regulation [21], and is implicated in the homologous recombination pathway for DNA repair [22].

Using various experimental approaches, we show that concerted, low-affinity interactions of the PSIP1-PWWP domain with both nucleosomal DNA and methylated histone tail result in specific and high-affinity binding to H3K36-methylated nucleosomes. During the final stages of our work, a similar conclusion was reached in another study [23]. Our study underscores this notion with a NMR analysis of the PWWP-nucleosome complex, a structural model of the complex based on experimental interaction data and an extensive *in vitro* and *in vivo* validation. Finally, based on a comparison with other PWWP domains and H3K36me-binding modules, we propose that the bipartite recognition of methylated histone tail and nucleosomal DNA is a general feature of H3K36me recognition.

## Results and discussion

### H3K36 methylation-dependent nucleosome binding by PSIP1-PWWP

**Figure 1:**
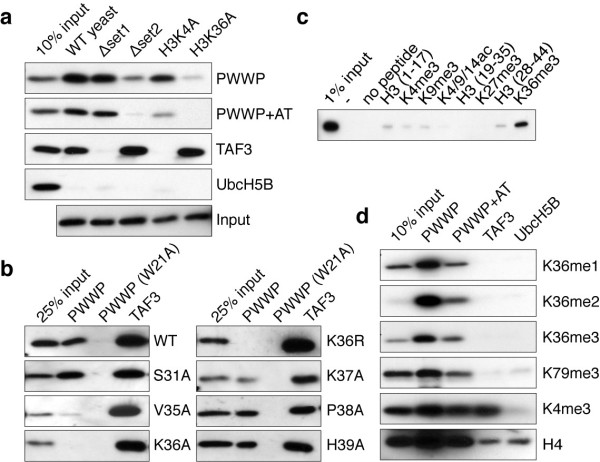
**H3K36 methylation-dependent nucleosome binding by the PSIP1-PWWP domain. (a)** Immunoblot analysis of GST pull-downs with the indicated GST-fusion proteins incubated with mono-nucleosomes extracted from (mutant) yeast strains probed for histone H3. **(b)** GST pull-downs with nucleosomes isolated from indicated (mutant) yeast strains. **(c)** Immunoblot analysis of pull-downs with GST-PWWP lysates and the indicated biotinylated histone H3 peptides probed for GST. **(d)** GST pull-down assay for GST-PWWP (PWWP), GST-PWWP including flanking AT-hook (PWWP+AT) or control proteins (TAF3, UbcH5B) were incubated with HeLa nucleosomes. Eluted proteins were detected by immunoblots with the indicated antibodies.

To characterize the interaction of the PSIP1-PWWP domain with H3K36me nucleosomes, binding assays with immobilized GST-PWWP and mono-nucleosomal fractions from wild type or mutant *Saccharomyces cerevisiae* were performed. Elimination of H3K36 methylation by deletion of the *SET2* histone methyltransferase gene or alanine substitutions of histone H3K36 strongly reduced the binding of nucleosomes to the PWWP domain (Figure 1a). In contrast, loss of H3K4 methylation in a Δ*set1* strain or a H3K4A substitution had no effect on PWWP binding but, as expected, eliminated the binding to the TAF3-PHD finger [24].

To determine the contribution of residues neighboring H3K36, mono-nucleosomes carrying point mutations for residues 31 to 39 in histone H3 were used. Of these only V35A, K36A and K36R affect PWWP-binding (Figure 1b), suggesting limited involvement of the H3 amino acid sequence around the K36 methylation site in determining binding specificity. The specific interaction of the PWWP domain with mono-, di-, and tri-methylated H3K36 was confirmed using biotinylated H3 tail peptides (Figure 1c) and mutation of W21 in the hydrophobic pocket of the domain completely abolished binding even in context of full-length PSIP1 (Additional file 1: Figure S1).

Next, immobilized GST-PWWP was used in binding to mono-nucleosomes prepared from mammalian cells. The bound fractions were enriched for H3K36me3 and H3K36me2 modifications, whereas they showed little enrichment for H3K79me3 and H3K4me3. Comparable results were obtained using an extended fragment of the PWWP domain including the flanking AT-hook region (Figure 1d) or full-length PSIP1 protein (Additional file 1: Figure S1b).

### Adjacent PWWP surfaces bind weakly to H3K36me3 peptides and DNA

**Figure 2:**
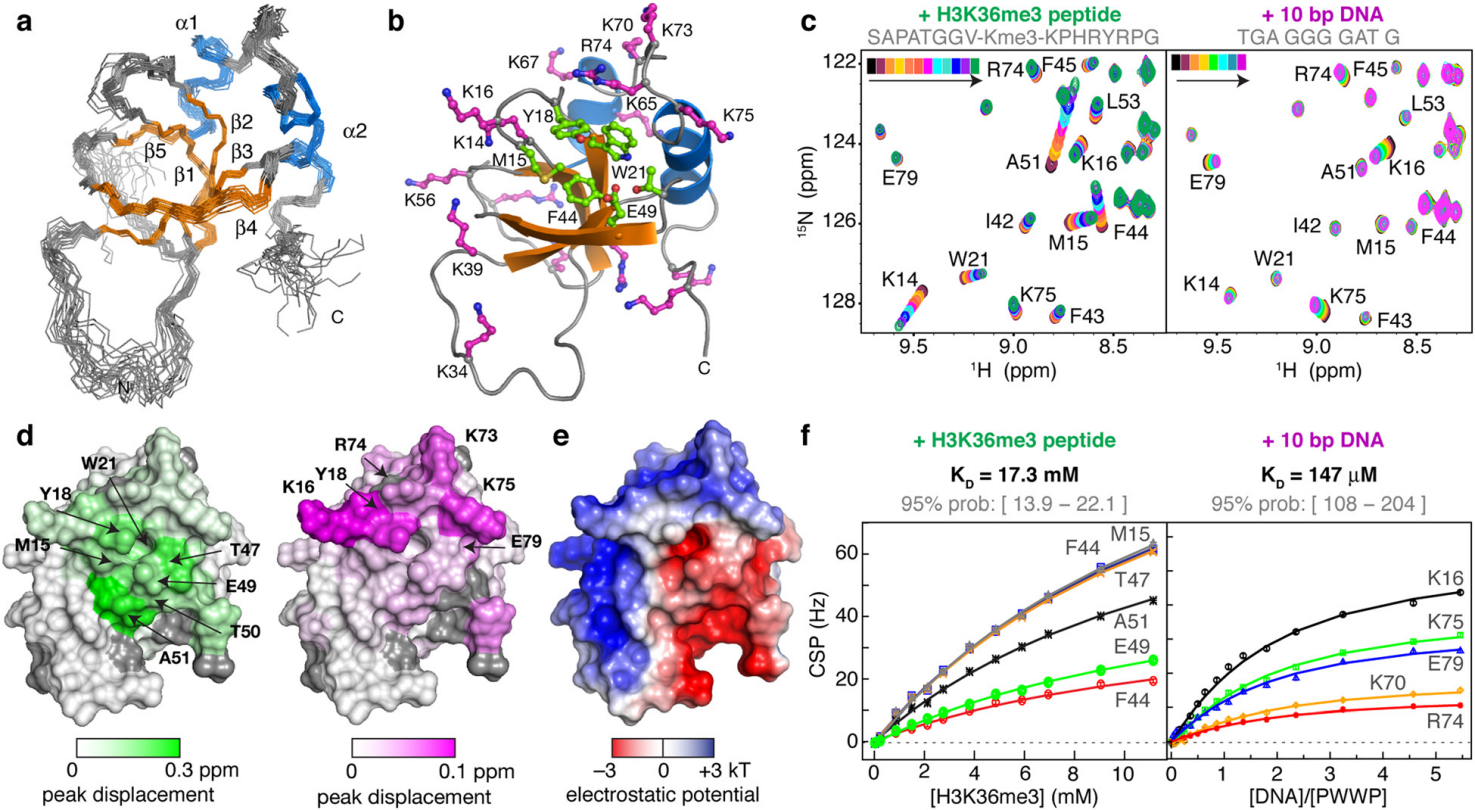
**Interaction with H3K36me3-methylated peptides and dsDNA. (a)** Ensemble of 20 best solution structures of PSIP1-PWWP domain; orange - β-sheet; blue - α-helix; gray - other. **(b)** Aromatic cage (green) and basic residues (magenta) shown as balls-and-sticks. Residues that have been mutated in this study are labeled. **(c)** Sections of the 2D ^1^H-^15^N HSQC spectrum of PWWP during the titration with H3K36me3 peptide (left panel) and dsDNA fragment (right panel). Free PWWP spectrum in black; resonances of interest are labeled. **(d)** Interaction surfaces for the histone peptide (left) and dsDNA (right), coded on the van der Waals surface. Grey is used for residues without data; residues with shifts larger than 10% trimmed mean + 2 σ are labeled. **(e)** Electrostatic potential on the solvent-accessible surface color **(f)** Chemical shift perturbation derived binding curves (symbols) including best fits (solid lines) for H3K36me3 peptide (left panel) and dsDNA (right panel).

In order to address the structural basis for its interaction with H3K36-methylated nucleosomes, we solved the solution structure of the PSIP1-PWWP domain (Figure 2a,b, Additional file 1: Figure S2 and Additional file 1: Table S1). As an HDGF-related PWWP domain [9], the core is formed by a five-stranded β-sheet core onto which two α-helices are packed. PSIP1-PWWP residues M15, Y18, W21, and F44 form an aromatic cage acceptor for a methylated lysine side chain. Notably, the surface of the PSIP1-PWWP domain is rich in basic residues that may interact with nucleosomal DNA (Figure 2b). As a first step in dissecting the contributions of histone tail and nucleosomal DNA in the PSIP1-PWWP-nucleosome interaction, NMR titration experiments were performed using a methylated histone peptide and a 10-bp DNA fragment.

Addition of a H3K36me3 peptide (residues 28 to 41) resulted in clear chemical shift changes for the backbone amide resonances of residues around the aromatic cage and strand β4 of the PWWP domain (Figure 2c,d left panels). The affinity for the H3K36me3 peptide is very low with a K_D_ of 17 mM (Figure 2f left panel). In part, this may be due to the relatively closed conformation of the binding pocket when compared to crystal structures of related PWWP domains bound to methylated peptides (Additional file 1: Figure S2c). Strikingly, this very low-affinity interaction is completely dependent on the methylation of H3K36, as no changes in chemical shift upon addition of non-methylated H3K36 peptide were observed, even at 11 mM of peptide (Additional file 1: Figure S3).

Addition of a dsDNA fragment that was previously suggested as a substrate for PSIP1-PWWP [25] resulted in chemical shift changes for a distinct set residues localizing to a single basic patch on the PWWP surface (Figure 2c and d, right panel). A K_D_ of 150 ± 50 μM was determined, indicating a low binding affinity for DNA (Figure 2f, right panel). Imino-proton resonances of DNA base pairs did not change in chemical shift or relative intensity during the titration, suggesting that the interaction lacks sequence-specificity (Additional file 1: Figure S4).

The DNA and histone interaction surfaces of the PWWP domain are adjacent and overlap with areas of positive or negative potential, respectively (Figure 2e). This arrangement provides an excellent electrostatic match to the negative phosphate backbone of the DNA and positive histone tail in the context of the nucleosome.

### Aromatic cage and basic surface determine binding specificity and affinity

**Figure 3:**
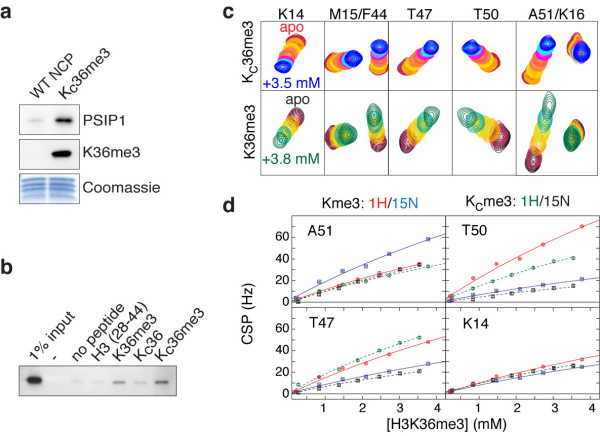
**H3KC36me3 MLA nucleosomes and peptides bind the PSIP1-PWWP domain with comparable affinities to their native counterparts (a) Recombinant MLA nucleosomes bind to endogenous PSIP1.** Nucleosomes were immobilized on beads using biotinylated 601 DNA and incubated with HeLa nuclear extract. Bound proteins were analyzed by immunoblot with the indicated antibodies and by Coomassie blue staining to control for the amount of histones. **(b)** PSIP1-PWWP binds preferentially to H3K36me3 and H3KC36me3 peptides. Indicated peptides were biotinylated and immobilized, incubated with GST-PWWP lysates and analyzed by immunoblot with GST antibodies. **(c)** H3K36me3 (lower panels) and H3KC36me3 peptides (upper panels) bind with similar affinity to same pocket in PSIP1-PWWP. An overlay of sections of the 2D ^1^H-^15^N HSQC spectrum is shown for each NMR titration point. Assignments of resonances of interest are indicated on top; color-coding is indicated in the figure. **(d)** Chemical shift perturbation (CSP)-derived binding curves and best fits for H3K36me3 and H3KC36me3 peptide. Only points up to 4 mM of peptide were included in the fit. Curves for a selection of residues are shown; color-coding is indicated in the figure.

In order to study the interaction of PSIP1-PWWP with H3K36me3 in the nucleosomal context, we made use of the methylated lysine analogue (MLA) approach [26], in which a cysteine is alkylated to result in a thioether mimic of the methylated lysine (referred to as H3K_C_36me3). Nucleosomes reconstituted from recombinantly expressed histones including H3K_C_36me3 are able to bind endogenous PSIP1 from a HeLa nuclear extract in a pull-down assay (Figure 3a). In a control experiment, a H3K_C_36me3 peptide and its native counterpart were found to bind the PSIP1-PWWP domain to a similar degree in a peptide pull-down assay (Figure 3b). Furthermore, comparison of NMR titration experiments of PWWP domain with either a native H3K36me3 or a H3K_C_36me3 peptide showed that peptide binding affects the same PWWP residues in a highly similar manner (Figure 3c). Based on the observed binding curve, we estimated a *K*_D_ value of 11 mM for the H3K_C_36me3 peptide (95% confidence interval: 6 <*K*_D_ < 26 mM), comparable to the *K*_D_ of the native peptide. This correspondence increases further when restricting the fit to the same titration interval, in which case the best-fit *K*_D_ is 13 mM for the native H3K36me3 peptide (95% confidence interval: 9 <*K*_D_ < 22 mM) (Figure 3d).

**Figure 4:**
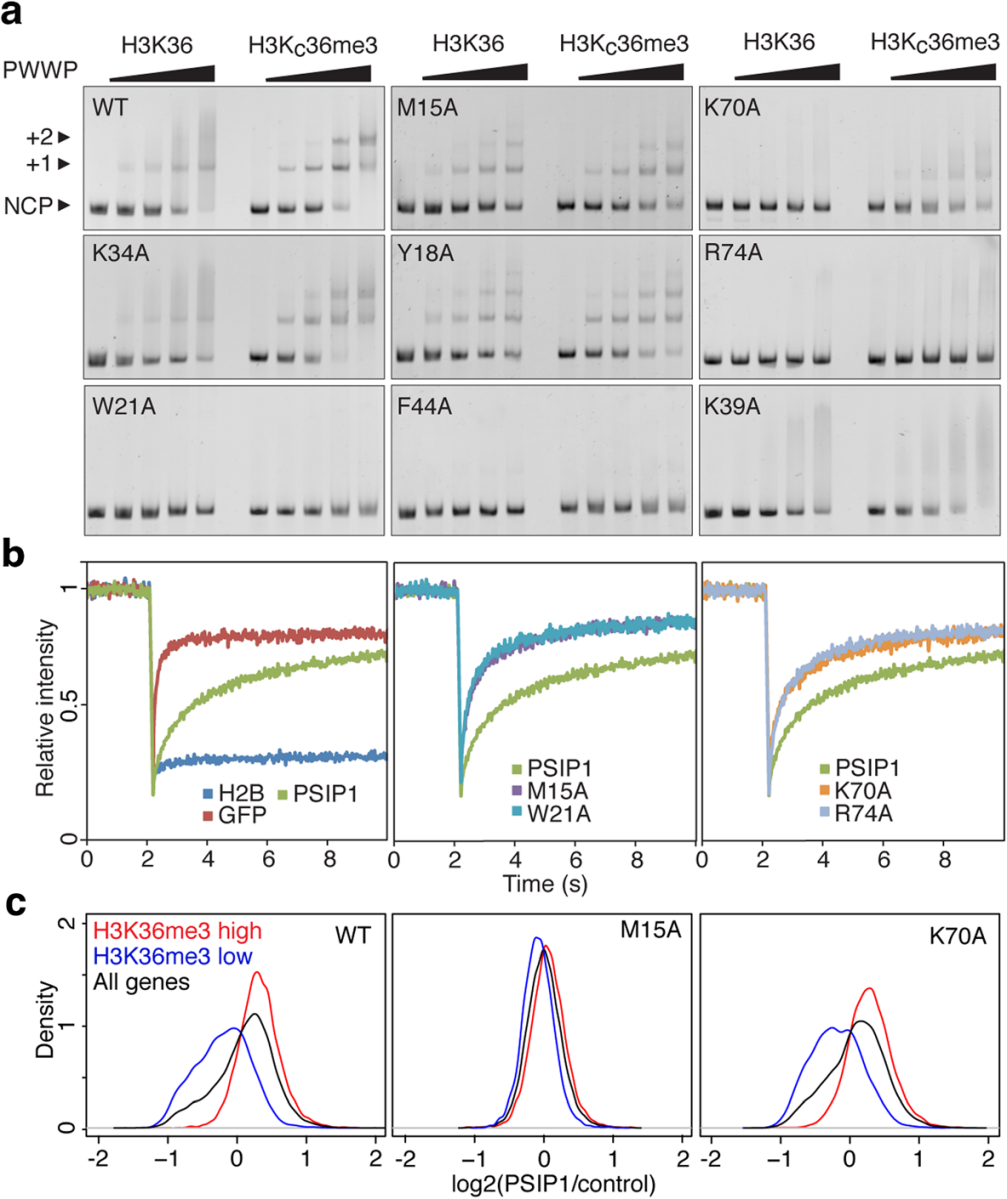
**Importance of the aromatic cage and basic surface of the PWWP domain in binding affinity and specificity. (a)** Titration of non-modified (H3K36) or modified nucleosomes (H3K36_C_me3) with 0, 0.5, 1, 2 and 3 molar equivalents of the indicated GST-PWWP protein added and analyzed by staining EMSA gels with ethidium bromide. Free nucleosome particles (NCP) or complexes with either one (+1) or two (+2) PWWPs, and the relevant PWWP mutation are indicated. **(b)** FRAP-recovery curves for GFP-PSIP1, indicated mutants or controls H2B-GFP or NLS-GFP. An average of 10 cells is presented. **(c)** Density plots are shown for the ratios PSIP1 (or mutant)/control for all genes (black), genes enriched for H3K36me3 (red) and genes not enriched for H3K36me3 (blue).

Having established the validity of the MLA approach, an extensive mutational analysis of the PWWP domain was carried out to dissect the contributions of the histone tail and DNA interaction surfaces to the binding affinity and specificity for methylated nucleosomes using electrophoretic mobility shift assays (EMSA). Wild type GST-PWWP preferentially binds to methylated over non-methylated nucleosomes (Figure 4a). A residual band shift observed in cases of non-modified nucleosomes suggests that PSIP1-PWWP can also associate non-specifically to nucleosomes, at least under the low ionic strength conditions of the EMSA experiment. Alanine substitutions of the aromatic cage residues M15 and Y18 strongly reduce specificity towards H3K_C_36me3 nucleosomes as shown from the similar patterns observed for modified and non-modified nucleosomes (Figure 4a). Notably, their effect on binding affinity is much less pronounced. Likewise, alanine mutation of two residues flanking the other side of the aromatic cage (H48 and E49) results predominantly in a loss of specificity (Additional file 1: Figure S5a). Alanine mutations of W21 and F44 that are both part of the aromatic cage showed no binding whatsoever (Figure 4a). These mutations may interfere with proper folding of the domain as they are part of the hydrophobic core of the protein and also suggested by their reduced soluble expression levels.

Mutations in the putative nucleosomal DNA-binding surface show a markedly different result. The K70A mutant binds with a lower affinity to nucleosomes, but retains preference for the modified nucleosomes. Strikingly, alanine substitutions of the solvent exposed R74 abolished the interaction with nucleosomes (Figure 4a). In contrast, the K34A mutant showed a comparable binding pattern to wild type PWWP (Figure 4a). Nearly all charge mutants showed severely reduced binding, but retained specificity for H3KC36me3 nucleosomes (Additional file 1: Figure S5a). Of these, K39 (shown in Figure 4a) and K56 were initially not found to interact with a DNA fragment in the NMR titration experiment, suggesting that binding to nucleosomal DNA involves a larger interaction surface.

To investigate the *in vivo* relevance of our findings, we examined the contribution of the PWWP domain to the mobility and distribution of PSIP1 in cells. To this end wt and mutant PWWPs were introduced in the context of full-length PSIP1 fused to GFP and expressed in HeLa cells (Additional file 1: Figure S6). The nuclear mobility of the GFP-PSIP1 proteins was measured by fluorescence recovery after photobleaching (FRAP). HeLa cells expressing NLS-GFP or histone H2B-GFP were included as highly mobile and immobile controls, respectively (Figure 4b left panel). FRAP curves for GFP-PSIP1 are indicative of transient chromatin-binding (Figure 4b green curve), consistent with previously published results [27]. Disruption of domain integrity (W21A), as well as mutations in the aromatic cage (M15A) or the DNA interaction surface (K70A, R74A), resulted in faster recovery of fluorescence after bleaching (Figure 4b center and right panels), demonstrating the requirement of both DNA and histone tail interaction surfaces for stable association with chromatin.

To examine the effect of PWWP mutations on the genomic distribution of PSIP1, the GFP-PSIP1 cell lines were used for chromatin immunoprecipitation followed by high-throughput sequencing (ChIPseq). To correlate binding of the PSIP1 proteins to H3K36 methylation, genes were divided into two groups: high H3K36me3- (red) or low H3K36me3- (blue) containing genes (Additional file 1: Figure S7). Wild type PSIP1 protein was selectively enriched on high H3K36me3 genes (Figure 4c and Additional file 1: Figure S7b), while aromatic cage mutant M15A showed no enrichment, in accordance with our *in vitro* data. Mutation of K70 did not significantly affect the genomic distribution of PSIP1 in correspondence with the mild effect on the affinity for nucleosomes of this mutant in EMSA.

### Concerted binding of methylated histone tail and nucleosomal DNA

The PWWP-H3K_C_36me3 nucleosome interaction was further analyzed using state-of-the-art solution NMR tailored for large supramolecular complexes. Recently, it was shown that a comprehensive characterization of protein-nucleosome interactions can be obtained [28] using methyl-group based NMR (methyl-TROSY) [29], in which only the histone methyl groups of isoleucine, leucine and valine (ILV) residues are observed.

**Figure 5:**
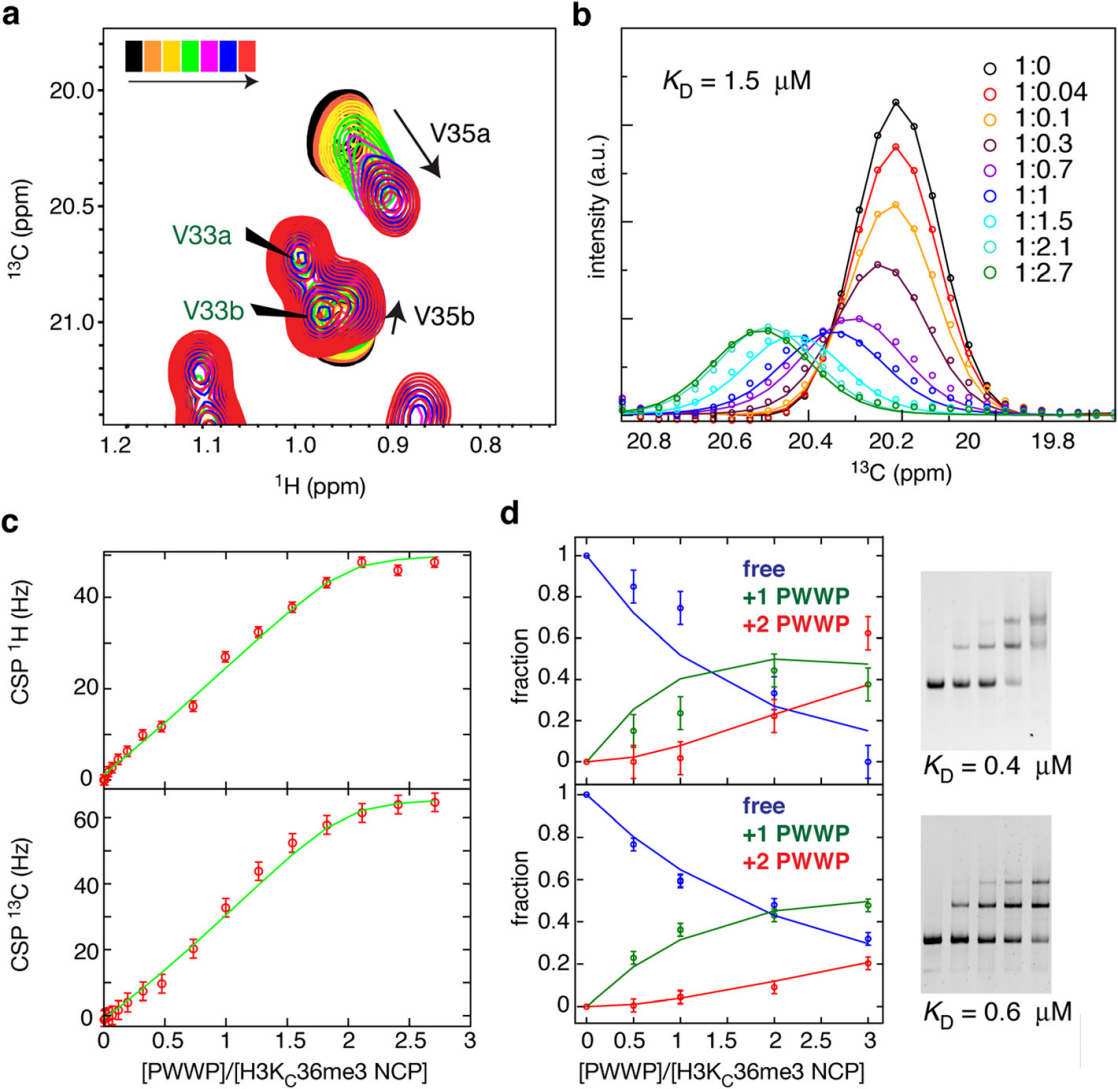
**Specific and high-affinity interaction of PSIP1-PWWP with H3K**_**C**_**36me3 nucleosomes. (a)** Sections of 2D ^13^C-^1^H methyl-TROSY spectra focusing on the resonances of the H3V35 methyl groups. Black spectrum corresponds to free nucleosomes and red to 1:2.7 molar ratio. PWWP ILV methyl groups are indicated with green labels. H3V35a/b refer to either of the V35 γ1/γ2 methyl groups. **(b)** Experimental (points) and fitted (solid lines) line shapes of H3V35a resonance, taken through the peak maximum in the ^13^C dimension, at the indicated molar ratios of nucleosome:PWWP. Best fit *K*_D_ is 1.5 μM (95% probability *K*_D_ < 8 μM) and *k*_off_ is 500 s^-1^ (95% probability 400 <*k*_off_ < 1000 s^-1^). **(c)** Line shape-derived binding curve, highlighting the saturation of the binding sites. Change in H3V35a peak position is plotted as a function of PWWP domain added; points - experimental values, lines - fitted values. **(d)** EMSA-based binding curves and fits for two independent titrations of H3K_C_36me3 nucleosomes with GST-PWWP (left), along with the ethidium-stained gels (right). Blue/green/red - integrated density of free/+1 PWWP/+2 PWWP nucleosome band. Estimated *K*_D_ values are indicated.

Here, we used MLA nucleosomes with ILV methyl-group-labeled histone H3 (spectrum in Additional file 1: Figure S8a) and monitored the H3V35 methyl groups to probe the PSIP1-PWWP interaction in a site-specific manner. Notably, the tri-methyl lysine mimic itself is not observable in these experiments as it is not isotope labeled. In the unbound state, the methyl group resonances of H3V35 (as well as H3L20) are very intense compared to those from the nucleosome core (Additional file 1: Figure S8b), reflecting the highly dynamic nature of the N-terminal tail. Addition of ILV-labeled PWWP domain resulted in a clear change in peak position of the methyl groups of H3V35, without affecting other methyl groups in the nucleosome core or the H3-tail (Figure 5a and Additional file 1: Figure S8a). Binding of PSIP1-PWWP also causes a local loss of flexibility of the H3 N-terminal tail around H3K36, as indicated by the sharp decrease in H3V35 peak intensity in the bound state, but not that of H3L20 (Figure 5b and Additional file 1: Figure S8b). For PSIP1-PWWP, the methyl group of I42, close to the aromatic cage, showed a distinct change in chemical shift during the titration experiment (Additional file 1: Figure S8a). Together, these changes demonstrate the specific interaction between the PSIP1-PWWP aromatic cage and the methylated H3 tail in the nucleosomal context.

Based on fitting the experimental line shapes of the H3V35a methyl group to a 1:2 (nucleosome:PWWP)-binding model, we find that the dissociation constant of PSIP1-PWWP-binding to the H3K_C_36me3 side chain within the nucleosome is 1.5 μM (Figure 5b, 5c and Additional file 1: Figure S9). The interaction is highly dynamic: the dissociation rate (*k*_off_) is ca. 500 s^-1^, corresponding to a lifetime of the complex (1/*k*_off_) of approximately 2 ms. The affinity found here at physiological ionic strength is comparable to the *K*_D_ value estimated from the gel-shift essay (ca. 0.5 μM), recorded at lower ionic strength and temperature (Figure 4d). Strikingly, the affinity of PSIP1-PWWP for methylated nucleosomes is four orders of magnitude higher than for a methylated peptide (*K*_D_ 17 mM) and two orders higher than for isolated DNA (*K*_D_ 150 μM). The enhanced affinity is due to simultaneous binding of both methylated histone tail (see chemical shift perturbations of H3V35, Figure 5b) and nucleosomal DNA (see the binding defects of DNA interaction surface mutants, Figure 4a). The magnitude of such enhancement in binding affinity upon linking of two binding sites cannot simply be predicted from the affinities for the isolated binding sites, as it depends crucially on the relative orientation of the linked sites and the length and flexibility of the linker [30,31]. Following the framework of Zhou [31], the enhancement may be expressed in the form of an effective concentration given by (*K*_*D*,*tail*_ × *K*_*D*,*DNA*_)/*K*_*D*,*nucleosome*_, which in our case evaluates to 1.5 × 10^− 4^ × 1.7 × 10^− 2^/1.5 × 10^− 6^ = 1.7 M. This enhancement value is significantly higher than typical values in the mM range as found for linked DNA-binding domains or bivalent pharmaceuticals [31,32]. This suggests that there are limited entropic losses and structural rearrangements upon binding. Thus, our data indicate that both the DNA and histone interaction surfaces of PSIP1-PWWP domain combine in a concerted manner to result in high-affinity binding to H3K36me3 nucleosomes.

### Structure of PSIP1-PWWP-H3K36me3 nucleosome complex

**Figure 6:**
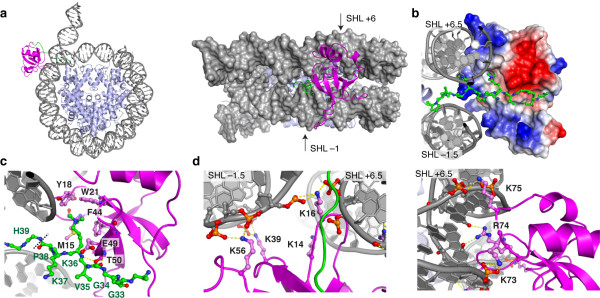
**Structural model of PSIP1-PWWP-H3K36me3 nucleosome complex. (a)** Lowest energy structure of the cluster that is in agreement with the cross-validation mutation data. **(b)** Detailed view showing H3 residues 31to 38 as balls-and-sticks, the DNA in cartoon representation and the van der Waals surface of the PWWP domain color coded by electrostatic potential. **(c, d)** Detailed view on the intermolecular interactions, focusing on the K36me3 recognition by the aromatic cage **(c)** and the DNA contacts mediated by K14, K16, K39 and K56 (**d**, left) and K73, R74 and K75 (**d**, right). Hydrogen bonds are shown as dashed yellow lines. Color-coding: magenta - PWWP domain, green - H3 tail, grey - DNA. PWWP residues are labeled in black, H3 residues in green. The dashed line in **(c)** indicates the peptide bond at which the H3 tail was cut in the flexible multi-domain docking protocol.

Our experimentally observed chemical shift perturbations and mutational analysis were used to derive a structural model for the PSIP1-PWWP-H3K36me3 nucleosome complex using the docking program HADDOCK [33]. In order to reliably sample a large conformational space for the flexible H3 tail, the flexible multi-domain docking protocol [34] was used. In addition, a large surface of DNA around the H3 tail exit site was systematically explored to sample all possible interaction sites on the nucleosomal DNA (Additional file 1: Figure S10). After clustering and cross-validation of the final ensemble of solutions (Additional file 1: Figure S11a, Additional file 1: Table S2), we find one cluster of solutions that is in agreement with the cross-validation data, with the lowest energy structure shown in Figure 6a. The PWWP domain bridges the two DNA gyres around super helical location (SHL) -1/+6, right at the H3 tail entry/exit site. There is an excellent electrostatic match with the nucleosomal DNA, while at the same time the H3K36me3 side chain is snugly captured by the aromatic cage (Figure 6b,c). Notably, the H3V35 methyl groups are more than 6 Å away from ILV methyl groups in the PWWP domain in the model, consistent with the absence of intermolecular NOEs (data not shown).

PWWP-DNA contacts made by the basic residues are mainly to the phosphate or sugar backbone (Figure 6c). The sequence-specific contacts of residue R74 seen in the lowest energy-structure are not conserved in the cluster of solutions. Overall, residues K73, R74 and K75 bind the DNA non-specifically around SHL +6, while K39 and K56 interact with the other DNA gyre at SHL −1. Residue K16 and K14 sit in between the two gyres and can interact with either. Notably, residues K67 and K70 do not mediate intermolecular interactions in many structures of this cluster, which may reflect the relatively minor binding defects of their alanine mutants in EMSA (see also Additional file 1: Table S2).

## Conclusions

Here we determined the molecular basis of H3K36me nucleosome recognition by the PSIP1-PWWP domain. We show that the interaction with nucleosomal DNA is responsible for an approximately 10,000-fold enhancement in binding affinity into an *in vivo* relevant range. A similar conclusion was reached in the work of Eidahl *et al*. from a comparison of an NMR- based estimated binding affinity for H3K36me3 peptides and a pull-down assay-based measurement of the affinity for H3K36_C_me3 nucleosomes [23]. While full length PSIP1 contains additional DNA binding domains [16,35], disruption of the PWWP basic surface markedly reduces *in vivo* chromatin binding ability of full-length PSIP1 as shown in this work and previously [27]. Moreover, mutations in the DNA interaction surface were previously shown to result in a dramatically reduced HIV-infectivity in cells [36], underscoring the functional significance of the bipartite nucleosome-binding for HIV integration and other PSIP1-dependent cellular processes.

**Figure 7:**
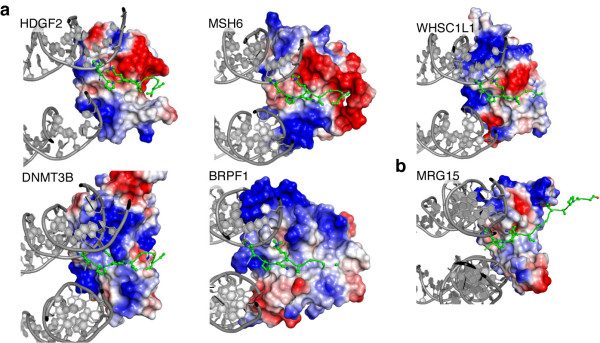
**Conservation of bipartite recognition of H3K36me nucleosomes. (a)** The PWWP domains of HDGF2 (3QBY), MSH6 (2GFU), DNMT3B (1KHC), BRPF1 (2X4X) and WHSC1L1 (2DAQ) were aligned with the PSIP1-PWWP domain in our model of the PWWP-H3K36me3 nucleosome complex. Detailed view showing H3 residues 31 to 38 as balls-and-sticks, the DNA in cartoon representation and the van der Waals surface of the PWWP domain color coded by electrostatic potential. **(b)** The chromodomain of MRG15 (2F5K) was manually docked on the H3K36me3 nucleosome model, positioning the aromatic cage around H3K36me3 and orienting its basic patches towards the DNA, while minimizing interatomic clashes.

Most H3K36me3-binding proteins depend on a PWWP domain for proper chromatin binding, despite their low affinity for methylated peptides. Therefore, nucleosomal DNA-binding may be a general driving force for the recognition of H3K36me3 nucleosomes *in vivo*. Superposition of homologous PWWP domains onto the structural model of the complex shows that these domains all share similar configurations of an aromatic cage and basic patches that potentially enable concerted binding to both methylated H3K36 side chain and nucleosomal DNA (Figure 7a). This holds true both for the close homolog HDGF2-PWWP domain, and more distantly related PWWP domains of MSH6, DNMT3b, BRPF1 and WHSC1L1. These proteins are involved in the DNA damage response, DNA and histone methylation and acetylation. Moreover, the bipartite binding mode observed for PSIP1 may extend beyond PWWP domains to other domains that bind H3K36me as well as to the recognition of other histone modifications close to the nucleosomal DNA, such as H4K20me. The MRG15 chromodomain that binds H3K36me also shows appropriately configuration of an aromatic cage and basic regions (Figure 7b). Similarly, another chromodomain and PWWP domain that bind H4K20me have been shown to have a distinct basic binding surface for DNA [37,38]. In contrast, PHD finger domains that specifically bind to H3K4me3 at the N-terminus of H3, have an extensive negative surface potential [39] and are therefore likely repelled from the nucleosomal DNA.

In conclusion, we propose that recognition of H3K36-methylated chromatin not only occurs through the methylated lysine side chain and its amino acid sequence context, but also through the nucleosomal DNA. We propose that this mechanism also applies to the recognition of other modifications close to the nucleosome core, such as H4K20me and H3K79me. This mechanism testifies to the fact that recognition of histone modifications relies on the binding to modified histone residues embedded in the chromatin fiber. Just as histones are not merely packaging material in chromatin, the nucleosomal DNA is not inert in the readout of the epigenetic modifications.

## Methods

### Protein expression and purification

The PWWP domain of human PSIP1 (3 to 100) or the PWWP+AT (3 to 207) were expressed as GST-fusions in BL21-DE3 or Rosetta 2 bacterial strains at 37°C in either LB medium or M9 minimal medium with ^15^NH_4_Cl and/or ^13^C-glucose. The protein was purified by binding to a glutathione agarose (GA) column (Sigma-Aldrich, St. Louis, MO) and eluted with 50 mM reduced glutathione (Sigma-Aldrich, St. Louis, MO). After thrombin digestion, PWWP was purified over a Sephadex-75 (HiLoad 16/60, GE Healthcare, Uppsale, Sweden) column in buffer A (50mM Tris-HCl pH 7, 100 mM KCl, 1 mM DTT, 1 mM EDTA, 0.5 mM PMSF and protease inhibitors), applied to a MonoS HR5/5 in buffer A and eluted using a linear gradient (0.1 to 1 M KCl).

*Drosophila* histones were expressed, purified and alkylated as previously described [26,40]. Histones used for NMR studies were produced in M9 minimal medium containing desired isotopes. Methyl-labeling of Ile-δ1-[^13^CH_3_] and Val/Leu-[^13^CH_3_,^12^CD_3_] (ILV-labeling) followed the procedure of Tugarinov [29].

### Antibodies and plasmids

α-GST (SC), α-PSIP1 (A300-848A, Bethyl, Montgomery, TX), α-H4 (07 to 108, Upstate), α-H3K36me2 (9758, Cellsignaling), α-H3K4me3 (ab8580,), α-H3K79me3 (ab2621), α-H3K36me1 (ab9048), α-H3K36me3 (ab9050; all “ab” antibodies obtained from Abcam, Cambridge, UK) and α-GFP (gift from Geert Kops) were used for ChIP and immunoblotting.

All GST fusions were cloned into pRPN265NB. PSIP1 cDNA was introduced into pEGFP-C using the Gateway system (Invitrogen, Carlsbad, CA). All point mutations were created using site directed mutagenesis (Stratagene, Santa Clara, CA). Stable GFP-tagged PSIP1 HeLa lines were created by cloning PSIP1 into pCDNA.5/FRT/TO (Invitrogen, Carlsbad, CA) and subsequent recombination into HeLa FRT cells carrying the Tet repressor for inducible expression [41].

### Nucleosome and peptide pull-downs

Mono-nucleosomes were extracted from HeLa or yeast cells by MNase treatment of lysed cells as previously described [24]. Histone H3 mutants were selected from a mutant library [42]. GA beads (Sigma-Aldrich, St. Louis, MO) were covered with GST-fusion proteins, mixed with mono-nucleosomes and washed. Eluted proteins were analyzed by immunoblotting. HeLa mono-nucleosomes were incubated with premixed GFP-fusion protein and GFP binder beads (ChromoTek, Planegg-Martinsried, Germany) and analyzed in a similar way. Peptide pull-downs were performed as described previously [24].

### Nucleosome reconstitution and EMSA

The 601-DNA ‘Widom’ template was amplified using PCR, purified using anion exchange chromatography and used for reconstitution using salt-gradient deposition. Nucleosomes were incubated with GA purified GST-PWWP protein in 0.2X TBE and analyzed by native 5% 60:1 acryl-bis gel electrophoresis. Either 1.5 or 3 pmol of nucleosome was used in all experiments and up to 3 molar equivalents of protein in 8 μL load volume. All steps were performed at 4°C. Gels were stained with ethidium-bromide and analyzed on a Gel-Doc XR+ system (Bio-Rad, Hercules, CA). If applicable, band densities corresponding to free, singly and doubly bound nucleosomes were quantified using ImageJ software package and subsequently fitted together to a 2:1 binding model using in-house written MatLAB routine (MATLAB version 7.13.0, The MathWorks Inc., Natick, MA).

### Strip-FRAP

FRAP studies were performed using a Zeiss 510 META confocal LSM (Zeiss, Oberkocken, Germany) as previously described [43]. GFP protein expression was induced with 0.5 μg/ml doxycycline for five hours.

### Chromatin immunoprecipitation

Chromatin preparation and ChIP were essentially performed as described [43,44]. Libraries were sequenced on AB/SOLiD 5500XL, producing 48 bp reads. Sequencing reads were mapped with Burrows-Wheeler Aligner (BWA-0.5.8c) (settings: -c -l 25 -k 2 -n 10) [45]. As a gene set, the known protein-coding genes as annotated in Ensembl 67 were used (http://www.ensembl.org). The number of reads mapped to each gene was normalized to the total number of reads mapping inside genes per sample. A separation of H3K36me3 enriched and non-enriched was made based on the density plot of the read density. Genes were filtered to have at least 50 sequencing reads in the GFP tagged PSIP1 ChIP-seq data. All plots were created using the R package (http://www.r-project.org/).

### NMR samples

Samples used for assignment and structure calculation contained ca. 1 mM PWWP domain in 90/10% H2O/D2O with 20 mM NaPi buffer at pH 6.2. Interaction studies were done at 0.3 mM PWWP in 20 mM NaPi pH 7.0 with 100 mM NaCl. ILV-labeled H3K_C_36me3 nucleosome sample contained 116 μM nucleosome in 20 mM NaPi pH 7 with 100 mM NaCl.

Peptides were extensively lyophilized and dissolved in NMR buffer to a stock concentration of 110 mM. Cysteine peptides were alkylated according to the MLA protocol [26] and purified using a Sephadex G-10 (GE Healthcare, Uppsale, Sweden) column followed by cation exchange chromatography. The purity of the peptide was confirmed by NMR. Annealed DNA oligos (Eurogentec, Liege, Belgium) were lyophilized and dissolved in NMR buffer to a stock concentration of 11.5 mM. Titration of H3K_C_36me3 nucleosomes was done using a PWWP stock of 1.28 mM.

### PSIP1-PWWP structure determination

NMR experiments for assignment, and structure calculation of the PSIP1-PWWP domain were carried out at 293K on a 600 or 750 MHz Bruker Avance II spectrometer (Bruker Biospin, Rheinstetten, Germany). Processing was done using the NMRPipe package [46]. Spectra were analyzed using Sparky (Goddard and Kneller, UCSF, USA). Backbone assignments were obtained using MARS [47] based on HNCACB and CBCACONH spectra. Side chain resonances were assigned using CCH-TOCSY, CBHD and NOESY spectra. Overall assignment completeness was 97.1% for all non-labile protons. Backbone dihedral angle restraints were derived using TALOS+ [48]. Distance restraints were derived from ^13^C- and ^15^N-edited 3D NOESY spectra (mixing time 120 ms). The NOE cross peaks were assigned and converted into distance restraints using CYANA 3.0 [49,50]. First, 10 ensembles of 100 structures were calculated by using CYANA using different random number seeds. Out of the 10 resulting distance restraint lists, only the restraints that were reproduced in all cases were retained to produce a final restraint list. This final list was then used to calculate 100 structures in CNS 1.2 [51], which were subsequently refined in explicit water by using the RECOORD protocol [52]. The final ensemble containing the 20 lowest-energy structures, contained neither distance violations > 0.5 A, nor dihedral angle violation > 5°, and was validated by using the iCing validation suite [53].

### Titration experiments and data analysis

Interaction studies of the PSIP1-PWWP domain were carried out at 293K on a 600 MHz Bruker Avance II spectrometer. Nucleosome spectra were recorded at 308K on a 900 MHz Bruker Avance III spectrometer with a TCI cryo-probe. Titration data were fitted using MatLAB scripts either using the fast-exchange assumption in case of fitting CSP derived binding curves or using explicit evaluation of the exchange matrix, and subsequent calculation of the FID in case of line shape fitting (see supporting materials in Kato *et al*. [28] for details).

### Molecular graphics

All molecular graphics were prepared using open-source PyMOL (The PyMOL Molecular Graphics System, Version 1.4, Schrödinger, LLC). Electrostatic surfaces were calculated using the adaptive Poisson-Boltzman solver [54] and the AMBER force field.

### Docking protocol PSIP1-PWWP–nucleosome complex

We used our experimental chemical shift perturbation, transferred-NOESY and mutagenesis data, together with available literature data to create a structural model for the PSIP1-PWWP-nucleosome complex with Haddock version 2.1 [33] and CNS 1.3 [51,55]. In what follows, we describe the docking procedure.

In short, the docking was divided in two stages: i) docking of the H3 N-terminal tail to the PSIP1-PWWP domain guided by the chemical shift perturbation, transferred-NOESY and mutation data, and using homology-derived interaction restraints from the homologous BRPF1-H3K36me3, HDGF2-H4K20me3/H3K79me3 crystal structures; ii) docking of the PWWP-H3K36me3 complex to the nucleosome, again guided by the chemical shift perturbation and mutation data, together with restraints to enforce the covalent attachment of the H3-tail to the remainder of H3. This approach was based on the flexible multi-domain docking protocol described by Karaca *et al*. [34]. It allows the efficient docking of the PSIP1-PWWP domain to both to K36me3 side chain in the H3 tail and to the nucleosomal DNA, and at the same time, to sample a large conformational space for the flexible H3 tail. This procedure is described in details in the Supplementary Material.

## Competing interests

The authors declare that they have no competing interests.

## Authors’ contributions

RvN carried out the biochemical and cell biology experiments, participated in its design and wrote the manuscript with HvI. RvS participated in the biochemical experiments. MS, SvH and EC participated in the ChIP-seq experiments and helped to draft the manuscript. RB participated in the design of the study and helped to draft the manuscript. MT and HvI conceived of the study, participated in its design and coordination and helped to draft the manuscript. HvI performed the NMR experiments, solved the structures and performed the modeling. All authors read and approved the final manuscript.

## PDB accession codes

The solution structure of the PSIP1-PWWP domain is accessible from the Protein Data Bank, [PDB: ID 3ZEH]. The structural model of the H3K36me3-nucleosome-PSIP1-PWWP domain complex is deposited under [PDB: ID 3ZH1] and is also available from the author’s website: http://www.nmr.chem.uu.nl/~hugo.

## Supplementary Material

Additional file 1: Supplementary Material. Click here for file
