# Supplementary material for: Nucleosomal DNA binding drives the recognition of H3K36-methylated nucleosomes by the PSIP1-PWWP domain
Source: Epigenetics Chromatin. 2013 May 8;6:12. doi: 10.1186/1756-8935-6-12 (PMC3663649; doi:10.1186/1756-8935-6-12)
Supplement: Additional file 1: — Supplementary Material. [file 1756-8935-6-12-S1.pdf]

Supplementary Material

Nucleosomal DNA binding drives the recognition of H3K36 methylated nucleosomes

Rick van Nuland[1], Frederik M.A. van Schaik[1], Marieke Simonis[2],
Sebastiaan van Heesch[2], Edwin Cuppen[2], Rolf Boelens[3],
H.Th. Marc Timmers[1] and Hugo van Ingen[3]

[1]Molecular Cancer Research, Division of Biomedical Genetics, University Medical Center Utrecht, Universiteitsweg 100, 3584 CG, Utrecht, The Netherlands.
[2]Genome Biology Group, Hubrecht Institute, Uppsalalaan 8, 3584 CT, Utrecht, The Netherlands.
[3]NMR Spectroscopy Research Group, Bijvoet Center for Biomolecular Research, Utrecht University Utrecht, Padualaan 8, 3854 CH, Utrecht, The Netherlands.

Correspondence should be addressed to H.v.I. (h.vaningen@uu.nl)

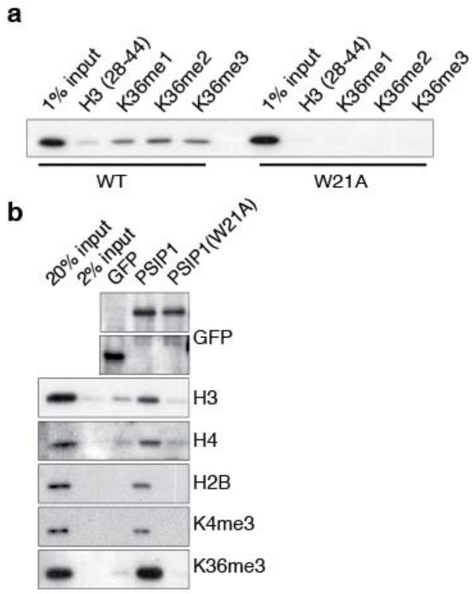

Supplementary Figure S1 PSIP1 binds mono-, di- and tri-methylated peptides dependent on the PWWP domain. (**a**) PSIP1-PWWP binds to H3K36me1, -me2 and -me3. Disruption of the PWWP domain abrogates the binding to H3 peptides. Immunoblot analysis of peptide pull-downs with GST-PWWP or W21A mutant lysates and the indicated peptides were probed with GST antibodies. (**b**) Full length PSIP1 enriched for H3K36 methylated nucleosomes and W21A mutation disrupts the interaction. GFP, PSIP1-GFP or the W21A mutant were transiently expressed in 293T cells and bound to beads and incubated with mono-nucleosomes extracted from HeLa cells. Bound fractions were analyzed by immunoblotting with the indicated antibodies.

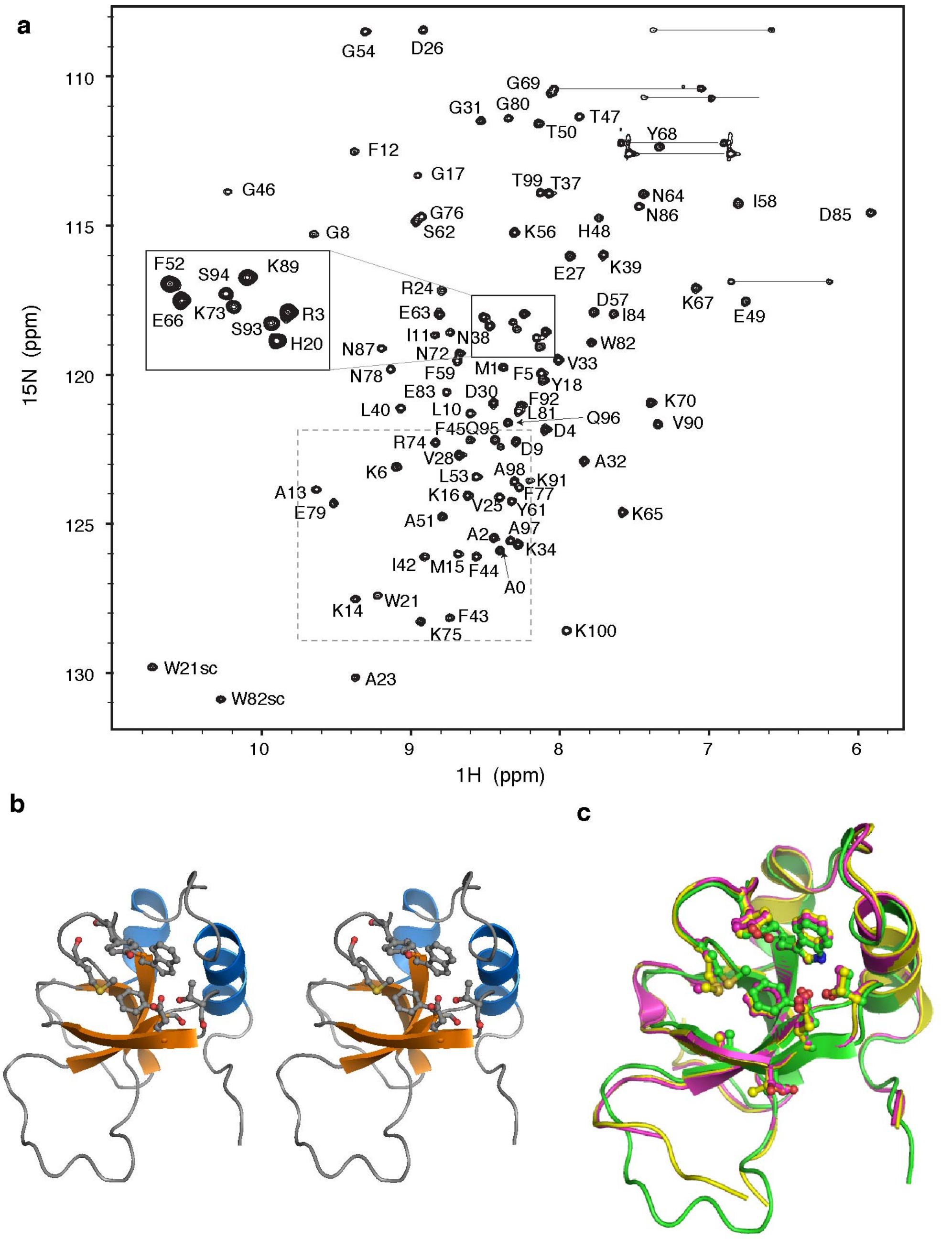

Supplementary Figure S2. Solution structure of PSIP1-PWWP domain. (**a**) Backbone assignments of the PSIP1-PWWP domain shown on the ^{15}N-^{1}H HSQC spectrum of free PWWP domain at 293K, pH 6.2, 600 MHz. The region in the solid box is expanded on the left; N and Q side chains resonances are indicated by the horizontal lines connecting the two resonances; indole side chain

resonances of W are indicated by the subscript ‘sc’. The region in the dotted box is shown in Figure 2 in the main text and Supplementary Figure S3. (**b**) Cross-eye stereo image of lowest energy structure; side chains of residues in the Kme3 binding pocket are shown as ball-and-stick. (**c**) Overlay of the unbound PSIP1-PWWP domain (green) and the HDGF2-PWWP domain in complex with H3K79me3 peptide (PDB: 3QJ6; yellow) and in complex with H4K20me3 peptide (PDB: 3QBY; magenta).

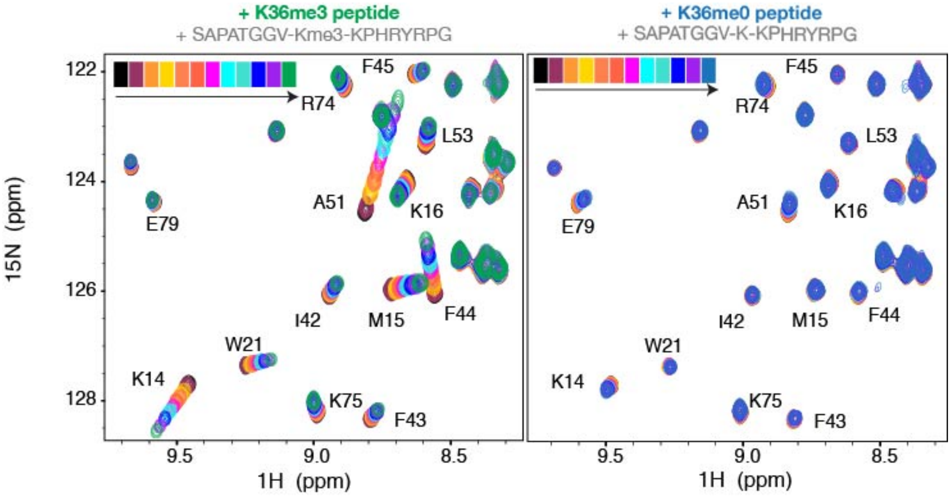

Supplementary Figure S3 Methylation of H3K36 is strictly required for the PSIP1-PWWP–H3 tail peptide interaction. NMR titration results for methylated (left) and non-methylated (right) H3K36 peptide, showing an overlay of the spectra for each titration point. Sequences of the fragments that were used for titration are indicated at the top. Color-coding of the spectra is indicated at the top, the free PWWP spectrum is in black. Final concentration of peptide was 11 mM in both cases (molar ratio PWWP:peptide is 1:27). Assignments of resonances of interest are indicated.

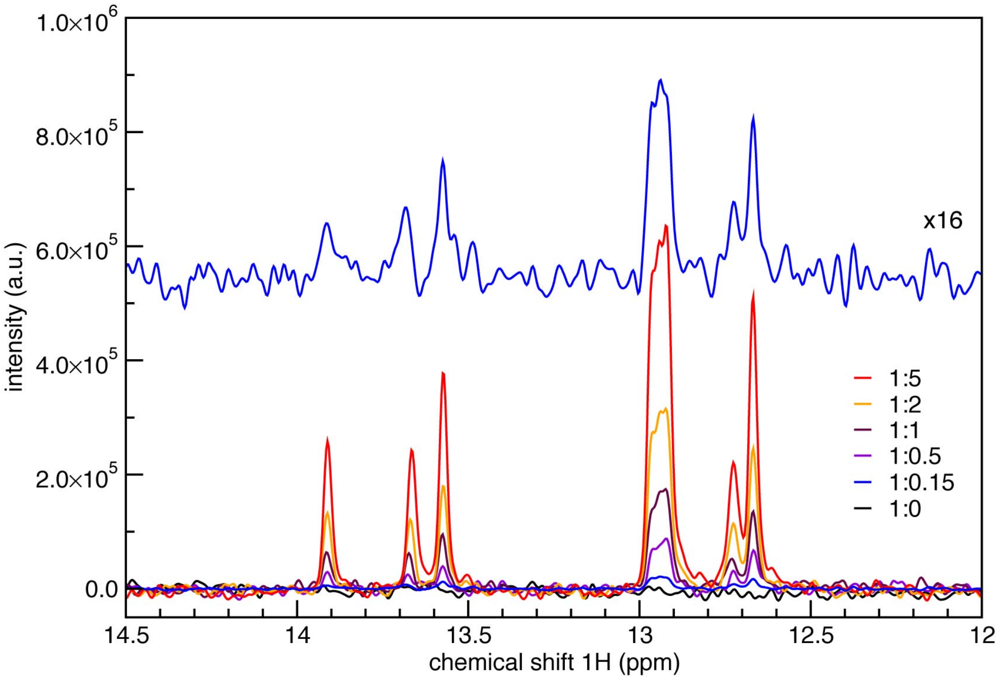

Supplementary Figure S4 PSIP1-PWWP interacts with DNA in a non-sequence specific manner. Overlay of 1D spectra (recorded at 600 MHz) taken during the titration of SRE-DNA fragment to PSIP1-PWWP domain, focusing on the DNA imino-protons. The protein:DNA molar ratio is indicated. In total 8 signals are visible from double-stranded DNA base pairs, which show no change in peak position, or relative intensity during the titration. The top spectrum in blue is amplified 16-fold to show that even at low protein:DNA ratio (1:0.15), where the DNA is mostly bound, the imino spectrum is the same as at high excess of DNA, where the DNA will be mostly free. This suggests that the PWWP-DNA interaction occurs through the phosphate backbone rather than through specific contact to DNA bases.

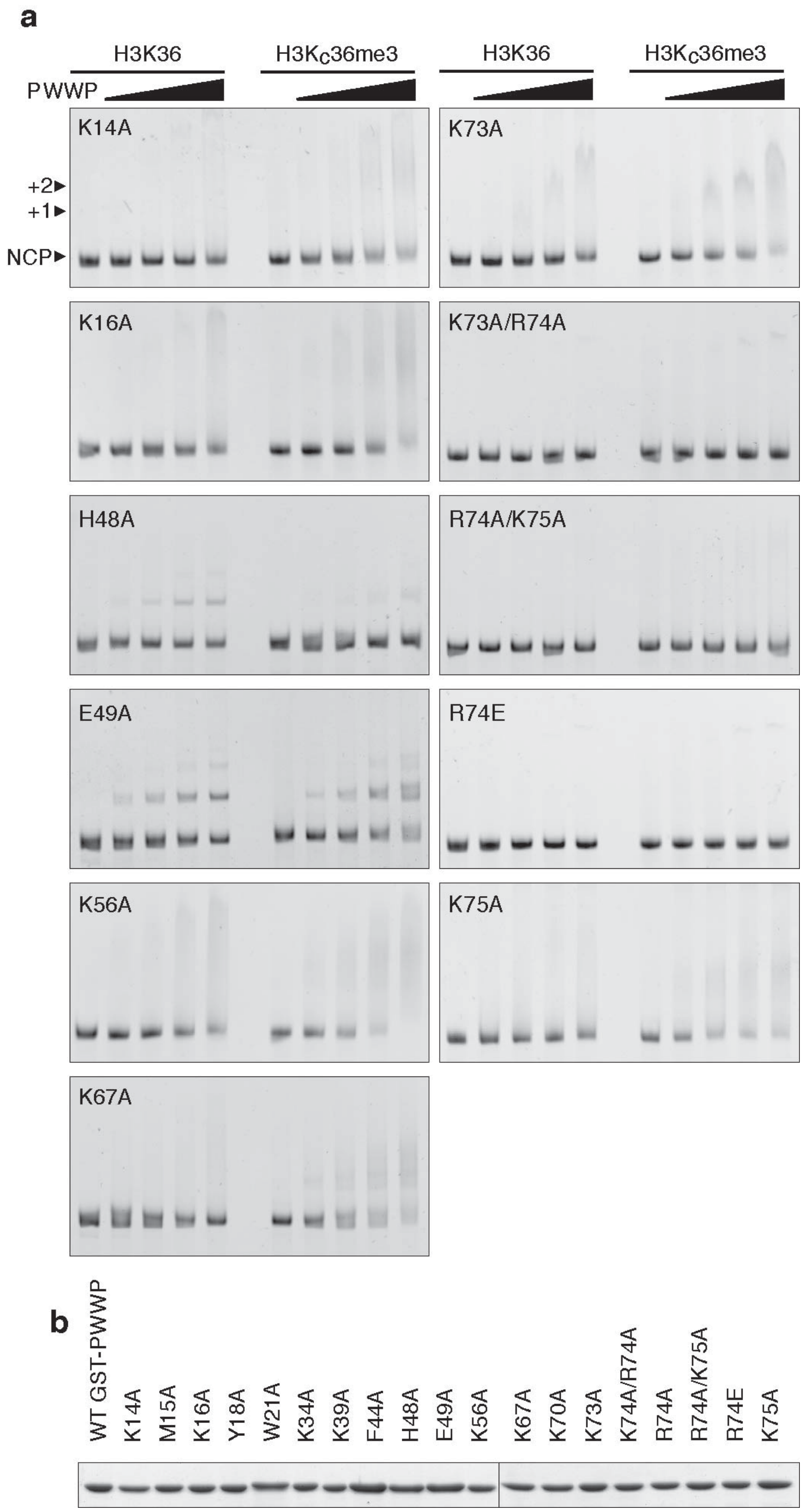

Supplementary Figure S5 Mutational analysis of PWWP aromatic cage and DNA interaction surface contribution to nucleosome binding affinity and specificity.

(**a**) Native gel electrophoresis with GST-PWWP and wild type or $H3K_c36me3$ nucleosomes visualized by staining the gel with ethidium bromide. Titration of GST-PWWP protein with indicated nucleosomes were incubated in a 1:0, 1:0.5, 1:1, 1:2 and 1:4 (nucleosome:PWWP) ratios and loaded on gel. Lower bands are free nucleosome core particles (NCPs). Upper bands are complexes of nucleosomes with either one (+1) or two (+2) GST-PWWP molecules. Each PWWP mutant is indicated on the gel. (**b**) Comparable amounts of single step purified GST-PWWP mutants were loaded on gel and used for EMSA. Proteins were visualized by coomassie blue staining.

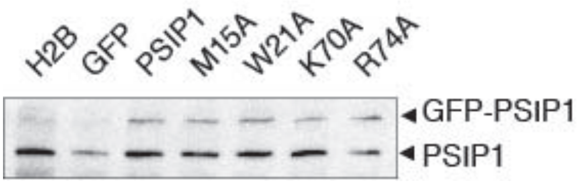

Supplementary Figure S6 Sub-endogenous PSIP1-GFP expression. Immunoblot analysis of HeLa FRT cells carrying PSIP1-GFP under a doxycycline-inducible promoter using PSIP1 antibodies. Cells were exposed to doxycycline for 4 hours, lysed in SDS sample buffer on gel. Lower band shows endogenous PSIP1 expression, upper band GFP-fusion protein. GFP-fusions are indicated above the blot.

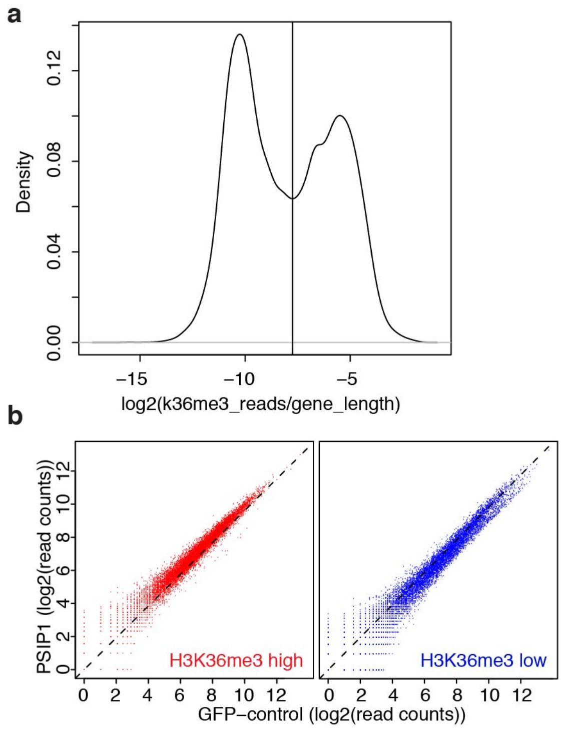

Supplementary Figure S7 (a) H3K36me3 read density in genes shows a bi-modal distribution. ChIP-seq was performed on H3K36me3. The number sequencing reads per gene was normalized to gene length (bp). The density plot of these normalized read counts is plotted. Genes are separated into H3K36me3 enriched and non-enriched using the minimum between the two peaks of the bimodal distribution as threshold (vertical line). (**b**) PSIP1 is enriched on H3K36 tri-methylated genes. Data points represents read counts per gene. Genes were divides in low (blue) and high (red) H3K36me3 signal.

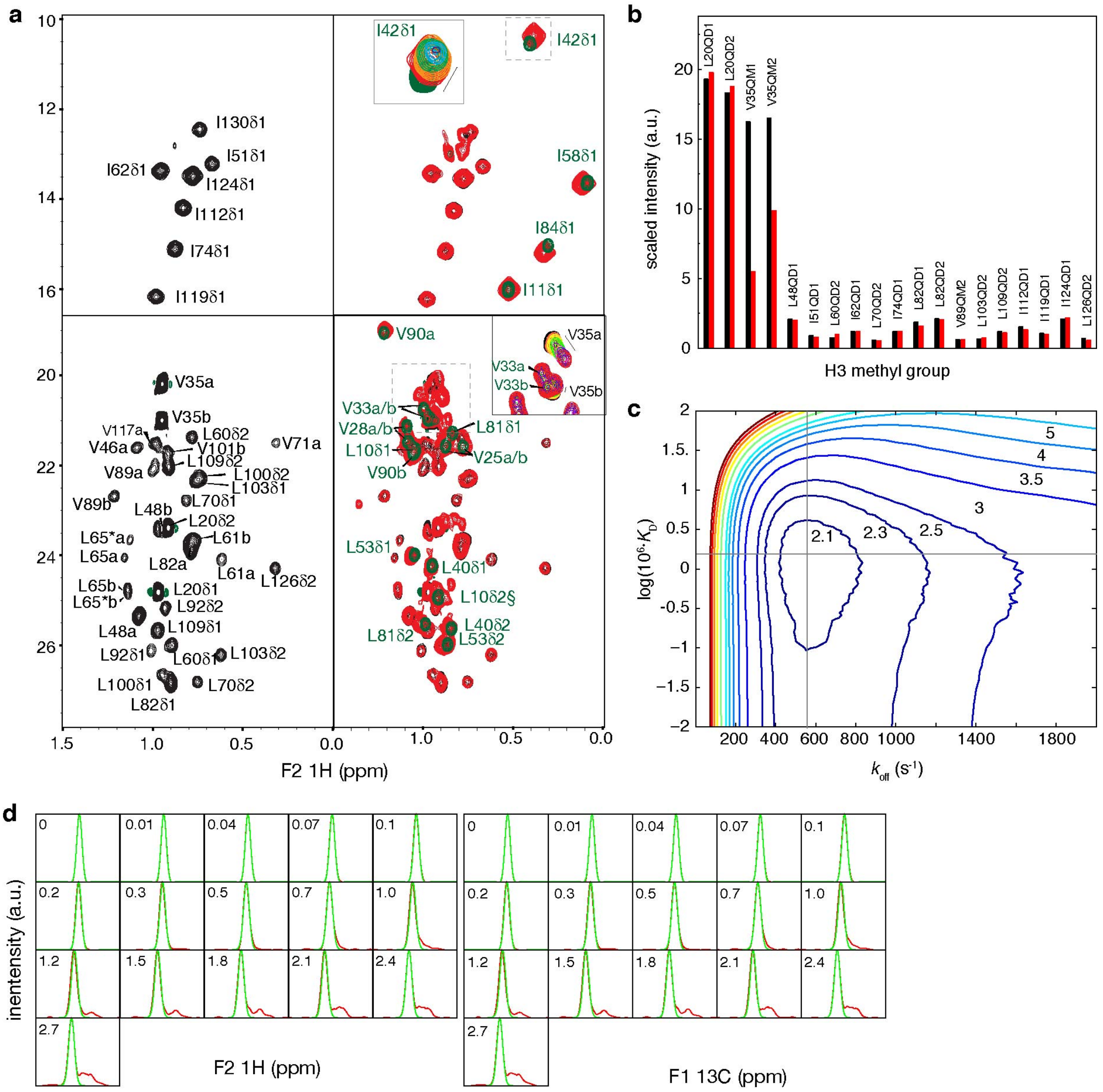

Supplementary Figure S8 Methyl-TROSY spectra of H3K$_C$36me3 nucleosomes in free and PWWP bound forms (recorded at 900 MHz). (**a**) Methyl-TROSY spectra of ILV labeled H3K$_C$36me3 nucleosomes in the apo state (black spectrum in left panel; including all H3 methyl group assignments); and overlay with PWWP bound state (red spectrum in right panel) and apo ILV-labeled PWWP (green spectrum in right panel). PWWP ILV residues are labeled in green in the overlay. The insets show an overlay of the titration spectra focusing on H3V35 and PWWP I42. Leu/Val methyl groups are labeled with their stereospecific assignments where available; otherwise the two methyl groups are arbitrarily

assigned as “a” or “b”. (**b**) Peak intensity of H3 methyl groups in unbound (black) and bound state (red); intensities are scaled such that median intensity for globular H3 residues is one. Interestingly, the peak intensity of V35 remains ~5-fold higher compared to the nucleosome core, which suggests the presence of residual dynamics within the complex. (**c**) Reduced χ^2-surface of line shape fitting of the H3V35a methyl group resonance as a function of k_{off} and K_D. Reduced χ^2 values for the contour line are indicated. Best-fit values are indicated by the intersection of the grey lines (χ^2_{red} = 1.99). F-statistic based critical value for 95% probability limit is 2.3. Data was fit to a 1:2 binding model, *i.e.* two PWWP molecules bind per nucleosome, one to each H3 tail, with each event independent from the other. Inclusion of cooperativity did not improve the quality of fit. (**d**) Individual line shape fits of the H3V35a resonance in the ^{1}H (left) and ^{13}C-dimension (right); molar equivalents of PWWP added are indicated; experimental line shape is shown in red, fitted line shape in green. The resonance is in fast-to-intermediate exchange ($\Delta\omega/k_{ex}$ = 0.4).

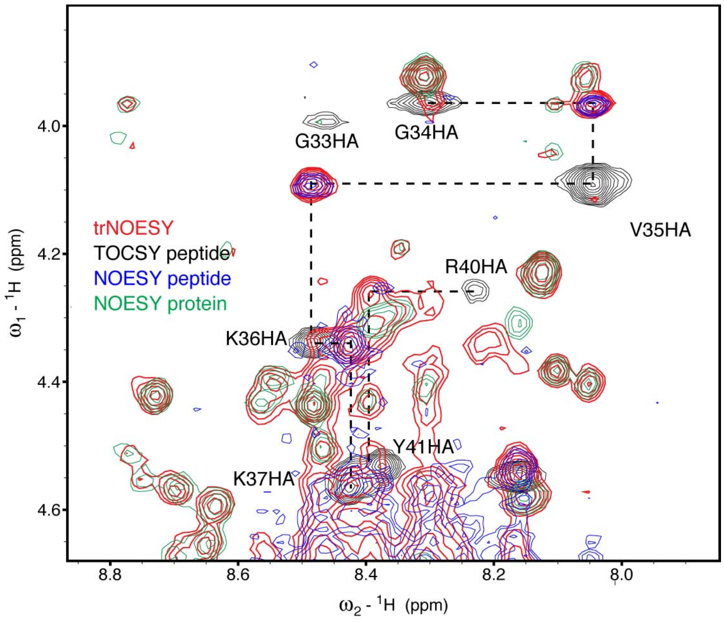

Supplementary Figure S9 Transferred-NOESY indicates an extended conformation for the H3 histone tail in complex with PSIP1-PWWP. Overlay of 2D ^{1}H-^{1}H transferred NOESY spectrum recorded on a mixture of 1 mM H3K36me3 peptide and 0.1 mM PWWP domain with 2D TOCSY and NOESY spectra recorded on isolated peptide or protein domain. All spectra were recorded in 20 mM NaPi pH 7 and 100 mM NaCl at 25°C at 500 MHz with 100ms mixing time. Strong αN connectivities in the transferred NOESY are observed for G34α-V35N, V35α-K36N, R40α-Y41N, as well as strong K37α-P38δ NOEs (not shown). These NOEs are consistent with an mostly extended conformation of residues 34-38 of the H3 peptide, as is also observed in crystal structures of methylated histones bound to PWWP domains[1, 2].

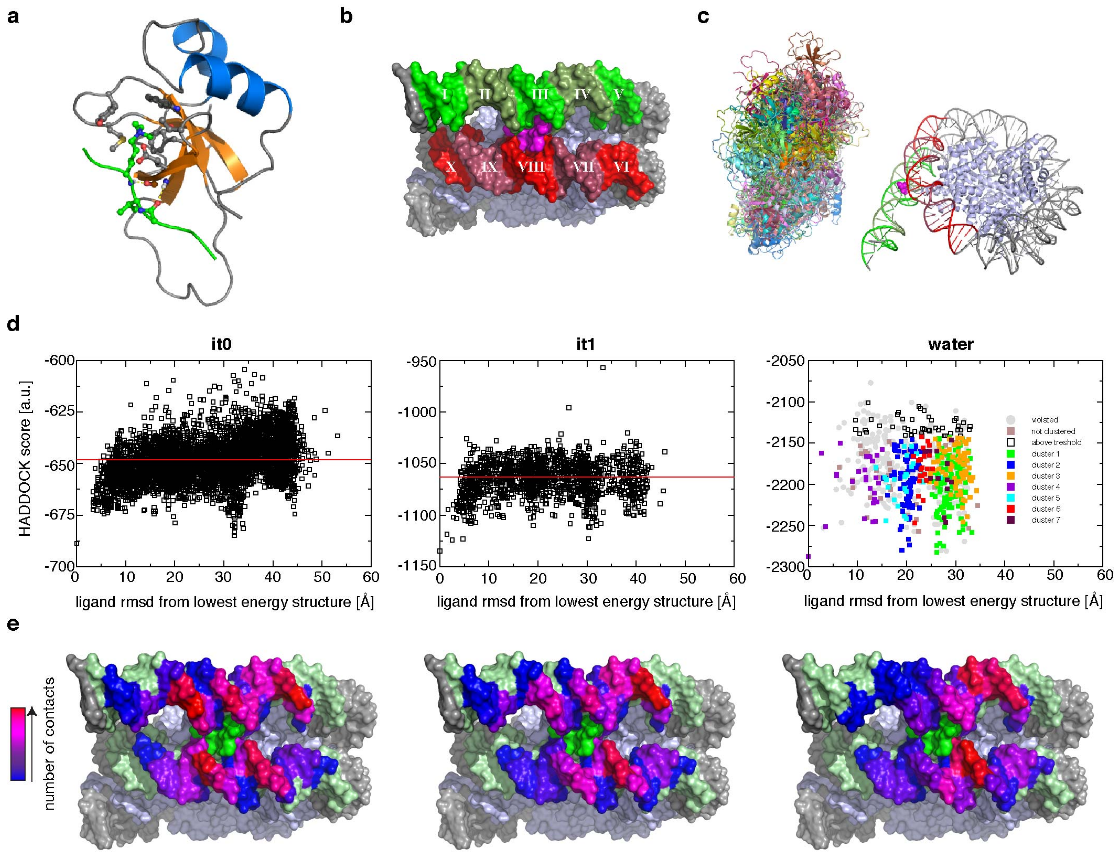

Supplementary Figure S10 Flexible multi-domain docking of PSIP1–H3K36me3 nucleosome complex. (**a**) Lowest energy structure of H3 tail residues 28-38 bound to PSIP1-PWWP domain obtained in the first docking stage. The aromatic cage residues of PWWP and residues G34-K36me3 of the H3 tail are shown in ball-and-sticks representation. The two enforced hydrogen bonds are indicated. (**b**) In the second stage the PWWP-H3K36me3 peptide complex is docked onto the nucleosome guided by DNA interaction restraints to one of the ten DNA patches indicated by roman numerals I-X and loose restraints to between the H3 peptide and the H3 tail exit site, residue 39 shown in magenta. (**c**) Overlay of the first 100 starting conformations of the PSIP1-PWWP domain relative to the nucleosome, in total 5500 random conformations were used. (**d, e**) Extent of conformational sampling during the second stage as judged by ligand rmsd (**d**)

and Arg74 mediated DNA contacts (**e**). HADDOCK score and ligand rmsd for all solutions after rigid body docking (it0, 5500 structures), semi-flexible refinement (it1, 1500 structures) and refinement in explicit water (water, 750 structures). For subsequent semi-flexible refinement 1500 structures of the best 3000 solutions in it0 (below the red line) were chosen, by skipping every other structure. For final waterrefinement the 750 best structures in it1 were chosen (below the red line). The final ensemble consist of all structures with a proper peptide bond across the former cut-site, without violations of the experimental restraints and with proper Ramachandran plot quality for the H3 tail (filled squares, 404 structures). (**e**) Contact analysis for residue R74 of PWWP for all solutions after it0, it1 and for the final ensemble. The DNA surface is color coded according to the number of contacts between R74 and each DNA nucleotide. The passive DNA surface is shown in light green.

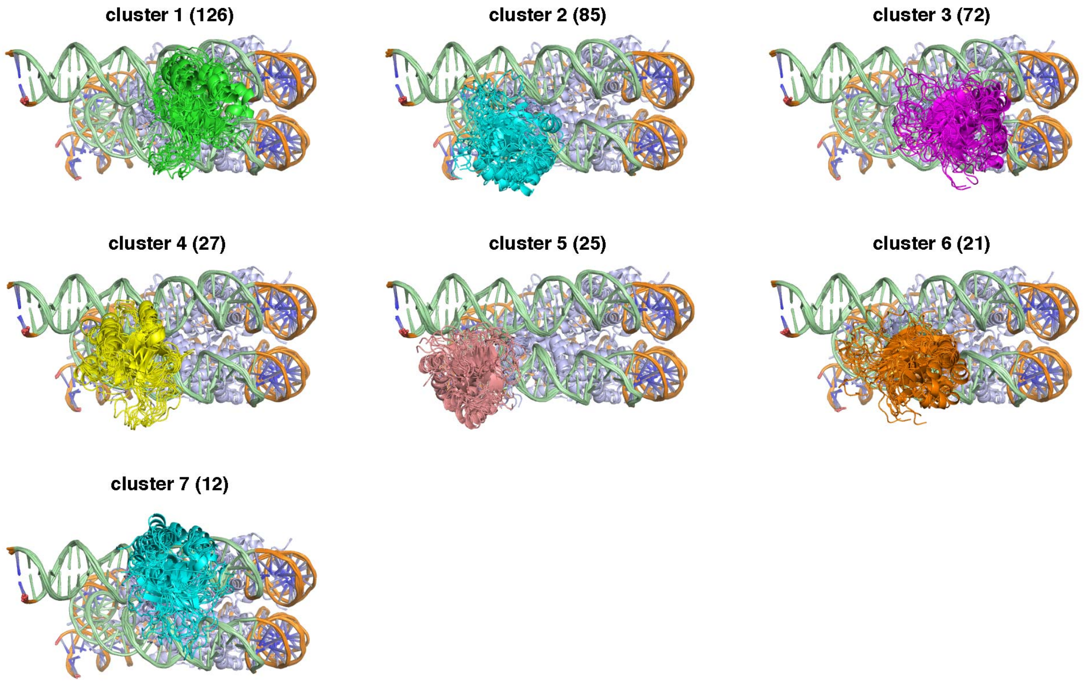

Supplementary Figure S11 Clustering analysis of final docking solutions. Superposition of the 10 best structures of for each of the 7 clusters found in the final ensemble of 404 solutions that are in agreement with experimental restraint data and have all backbone dihedral angles for residues 37-40 in H3 tail in core and allowed regions of the Ramachandran plot. Number of structures in each cluster is indicated between brackets.

Supplementary Table S1 Structural statistics for the free PSIP1-PWWP domain.

A. Restraint information	
number of experimental distance restraints	1988
intra-residual/sequential/medium/long	451/612/316/609
TALOS derived dihedral angle restraints ϕ/ψ	145
B. Average RMS deviation from experimental restraints	
All experimental distance restraints (Å)	0.017 ± 0.001
All dihedral angle restraints (°)	0.49 ± 0.05
C. Coordinate RMS deviation (Å)	
Average RMSD to mean	
Ordered backbone atoms	0.47 ± 0.30
Ordered heavy atoms	0.82 ± 0.51
Global backbone atoms	0.82 ± 0.75
Global all heavy atoms	1.19 ± 0.92
Pairwise RMSD	
Ordered backbone heavy atoms	0.85 ± 0.22
Ordered all heavy atoms	1.49 ± 0.23
Global backbone heavy atoms	1.67 ± 0.41
Global all heavy atoms	2.24 ± 0.42
D. iCing ROG score (red/orange/green %)	9/25/67
E. Ramachandran quality parameters (%)	
Residues in most favoured regions	84.2
Residues in allowed regions	15.2
Residues in additionally allowed regions	0.3
Residues in disallowed regions	0.2
G. Average RMS deviation from current reliable structures (null deviation=1)	
Bond lengths	1.16 ± 0.01
Bond angles	0.52 ± 0.02
Omega angle restraints	0.77 ±0.05
Side-chain planarity	1.06 ± 0.21
Improper dihedral distribution	0.90 ± 0.04
Inside/outside distribution	1.02 ± 0.02
H Average deviation from current reliable structures (Z-scores, null deviation = 0)	
2nd generation packing quality	+5.8 ± 2.0
Ramachandran plot appearance	-2.7 ± 0.4
Chi-1/Chi-2 rotamer normality	-2.2 ± 0.7
Backbone conformation	-1.3 ± 0.4

[a] statistics are given for residues 1-93 of PSIP1-PWWP, excluding the unstructured N-terminal cloning artifact. (residues -5 to 0) and the dynamically disordered C-terminal 7 residues (94-100) for which no NOEs were oberved. Ordered regions are residues 5-30 and 38-89 of the PWWP domain.

Supplementary Table S2 Residue-specific contribution to the intermolecular energy.

res.[a]	intermolecular energy (σ) in kcal/mol							g.s.[d]
	**cl. 1[b] #126**	**cl. 2 #85**	**cl. 3[c] #72**	**cl. 4[d] #27**	**cl. 5[d] #25**	**cl. 6[d] #21**	**cl. 7[d] #12**	
**K16**	**-147 (30)**	**-134 (26)**	-83 (40)	**-135 (31)**	**-114 (40)**	**-103 (45)**	-80 (37)	++
**K67**	-14 (31)	+1 (2)	-16 (35)	+1 (2)	-22 (37)	-4 (19)	-2 (4)	+
**K70**	-68 (36)	-51 (46)	-65 (54)	-45 (49)	-12 (28)	-86 (46)	-44 (48)	+
**K73**	-94 (46)	-77 (31)	-81 (37)	-81 (33)	-73 (34)	-62 (51)	**-102 (36)**	++
**R74**	-68 (31)	-80 (29)	-69 (34)	-95 (23)	-71 (26)	-99 (26)	-70 (23)	++
**K75**	-46 (41)	-92 (40)	**-97 (33)**	-83 (51)	-65 (49)	-61 (56)	-101 (42)	++
R3	-8 (27)	-	-	-	-8 (20)	-	-	n.d.
K6	-	-	-	-	-	-	-	n.d.
K14	-58 (52)	-80 (45)	-54 (51)	-48 (48)	**-29 (49)**	-38 (35)	-98 (23)	++
K34	-13 (36)	-10 (27)	-26 (43)	-7 (18)	-1 (2)	-3 (10)	-9 (31)	-
K39	-75 (54)	-60 (44)	**-13 (33)**	**-30 (41)**	-92 (46)	**-**	**-**	++
K56	-54 (44)	**-21 (43)**	**-10 (30)**	**-1 (3)**	-92 (40)	**-6 (21)**	**-20 (35)**	++
K65	-	-	-	-	-	-	-	n.d.
K91	-2 (4)	-1 (3)	-4 (16)	-1 (2)	-2 (3)	-1 (1)	-0 (2)	n.d.

a active residues are shown in bold and have been explicitly restrained to contact the DNA in 50% of the docking solutions. Other residues have not been restrained.

b number of structures in each cluster indicated with '#'. Largest contribution to the intermolecular energy is shown in bold. Cells are filled in dark/light green if the residue makes a favorable contact (average + 2/1σ < 0). Intermolecular energy contributions that conflict with the gel-shift based mutation data is shown in bold-red.

c based on all structures in the cluster, otherwise based on best 50.

d ++/+/- = strong/moderate/no loss of binding affinity based on gel-shift binding assay of alanine mutants (see Fig. 3 and Supplementary Fig. S6). n.d. is not determined.

Supplementary Methods

Docking protocol PSIP1-PWWP–nucleosome complex. We used our experimental chemical shift perturbation, transferred-NOESY and mutagenesis data, together with available literature data to create a structural model for the PSIP1-PWWP–nucleosome complex with Haddock version 2.1 [3, 4] and CNS 1.3 [5, 6]. In what follows, we describe the docking procedure.

In short, the docking was divided in two stages: i) docking of the H3 N-terminal tail to the PSIP1-PWWP domain guided by the chemical shift perturbation, transferred-NOESY and mutation data, and using homology derived interaction restraints from the homologous BRPF1-H3K36me3, HDGF2-H4K20me3/H3K79me3 crystal structures; ii) docking of the PWWP-H3K36me3 complex to the nucleosome, again guided by the chemical shift perturbation and mutation data, together with restraints to enforce the covalent attachment of the H3-tail to the remainder of H3. This approach was based on the flexible multi-domain docking protocol described by Karaca et al. [7, 8]. It allows to dock the PSIP1-PWWP domain efficiently to both to K36me3 side chain in the H3 tail and to the nucleosomal DNA, and at the same to sample a large conformational space for the flexible H3 tail.

First, residues 31-38 of the H3-tail with a native trimethylated K36 (M3L) were docked to the PSIP1-PWWP domain, using the solution NMR structure ensemble containing 20 structures. To prepare these structures for docking, side chain dihedral χ_3 of M15 was adjusted to match the corresponding dihedrals found in the H3K36me3 bound HDGF structures, resulting in a bound

conformation of the binding pocket. The resulting structures were subsequently re-refined in explicit solvent using the default HADDOCK protocol. Based on pH titration data, His48 was set to be singly protonated (data not shown). The proton was placed at Nε2 to optimize the hydrogen-bonding network. The PWWP domain was set to be semi-flexible. The H3-tail peptide structure was taken from the crystal structure of the homologous PWWP domain of BRPF1 bound to a H3K36me3 peptide (PDB-ID: 2X4X [1]), and was set to be fully flexible during docking. To increase sampling of the conformational space, the number of MD steps during the flexible simulated-annealing stage was doubled compared to their default values.

Docking was driven using ambiguous interaction restraints derived from chemical shift perturbation mapping and mutational analysis. Specifically, all PWWP residues with CSP larger than the 10% trimmed mean + 2σ in the me3-peptide titration and a surface accessible area of more than 33% or non-basic residues that led to loss of binding in the gel shift assay were defined as active resides. Neighboring solvent exposed residues were defined as passive residues. On the side of the H3 tail peptide only K36me3 was defined as an active residue and V35 and K37 were defined as passive residues. In total there were 12 ambiguous interaction restraints of which half were randomly removed for each calculated solution. In addition, ambiguous interaction restraints were defined between K36 methyl group protons and the aromatic cage residues of the PWWP domain (M15, Y18, W21 and F44), based on the total lack of chemical shift changes in the titration experiment using a non-methylated peptide. Backbone dihedral angle restraints consistent with an extended conformation (ϕ: -135±45°

/ ψ: +135±45°) were defined for residues 34-38 of the H3 peptide based on the transferred-NOESY data. Finally, homology based backbone hydrogen bond restraints were added between PWWP Thr50 and H3 tail K36 and G34. Equivalent hydrogen bonds are present in all BRPF1/HDGF PWWP complexes with a tri-methylated lysine peptide.

In the rigid body docking phase, 200 solutions were calculated, of which the best 100 structures according to their HADDOCK score were refined in the (semi-)flexible phase, and further refined in explicit solvent. The best 50 structures cluster within 4 Å heavy backbone atom interface rmsd for the ligand. The lowest energy structure is shown in Supplementary Figure S11a. All solutions were subsequently reduced to 11 representative structures using an all-atom RMSD based clustering with a cut-off of 2.6 Å, to be used as input for docking onto the nucleosome.

In the second stage, the PWWP-H3K36me3 complexes were docked onto the nucleosome guided by i) CSP and mutation derived ambiguous interaction restraints between basic residues of PWWP and the nucleosomal DNA around the H3-tail exit site; ii) restraints to enforce covalent attachment of the H3-tail in the PWWP complex to the remainder of H3 in the histone octamer. Docking was set up as a three-body docking, with separate chains for the nucleosomal DNA, the histone octamer with H3 up to residue 39, and the PWWP-H3K36me3 complex with H3 residues 28-38. The cut of histone H3 is at the point where it emerges from between the DNA helices, forming a natural point to allow for hinge like motions in the flexible multi-domain protocol. It should also be noted that the conformation of residue 38 and onwards in H3 are strictly conserved in

all nucleosome structures, allowing an unambiguous definition of this hinge point. The starting conformation of octamer and DNA were derived from the crystal structure of *Dm.* nucleosome, PDB 2PYO [9]. Note that this DNA sequence corresponds to the α-Satellite rather than the '601'-sequence used experimentally. Since recognition of DNA by both octamer and PSIP1-PWWP domain is not sequence-specific, this is not of concern. The DNA was further extended by adding a 10 bp straight B-DNA segment based on PDB-ID 355D and subsequent refinement. All lysine and arginine side chains in the PWWP domain were rebuilt by CNS, after removing all side chains atoms beyond the Cβ. This procedures breaks intra-molecular salt-brigdes within the PWWP domain, resulting exposed arginine and lysine side chains that are free to interact with DNA.

Ambiguous interaction restraints between PWWP and the nucleosomal DNA were derived from chemical shift perturbation mapping and mutational analysis. Specifically, basic PWWP residues were defined as active residues if i) their CSP was larger than the 10% trimmed mean + 2σ in the SRE-DNA titration, ii) their solvent accessible surface area of is more than 33%, and iii) their mutation resulted in a clear loss in binding affinity for K_C36me3 nucleosomes in the gel-shift essay. These criteria resulted in 6 active residues for the PWWP domain (K16, K67, K70, K73, R74 and K75). In the absence of experimental data on the interaction site on the nucleosomal DNA, we scanned across the DNA around the H3 tail exit site (+/- 12-bp) by defining 10 sets of passive residues for the DNA, each 5-bp in length (see Supplementary Fig. S11b). For every docking

solution calculated, ambiguous interaction restraints were defined between 3 randomly chosen active PWWP residues and one of the 10 passive DNA sets.

To ensure that the nucleosome remains intact during docking, the starting conformations were not randomized in the rigid body docking protocol. Rather, 5500 starting conformations were generated by randomly rotating the PWWP-H3K36me3 complex and placing it randomly in a box of dimensions 25x50x100 Å at ca. 50 Å from the nucleosome, oriented towards the H3 tail exit site (see Supplementary Fig. S11c).

In the rigid body docking phase, 5500 solutions were calculated, i.e. 100 per combination of PWWP-H3K36me3 input structure and set of passive DNA residues. To allow sampling of large conformation space, a loose 12 Å unambiguous restraint between Cα of K36 and Cα of H39 was defined. All solutions were ranked according to their HADDOCK score, which is a weighted sum of van der Waals, electrostatic, desolvation, binding and restraint energies, and the buried surface area.

The extent of conformational sampling was checked by calculating the ligand rmsd of all solutions, where ligand rmsd is defined as the heavy atom backbone rmsd of the PWWP domain when superimposing the structures on the histone octamer (Supplementary Fig. S11d). The very high ligand rmsd of up to 55 Å shows that the PWWP domain occupies different positions on the nucleosomal DNA as anticipated. Notably, there are structures with low (favorable) scores over the whole range of ligand rmsd.

In addition, all DNA contacts (heavy atom distance < 5 Å) made by basic PWWP residues were analyzed. Of all these residues, R74 makes the most

contacts. In 89% of the solutions, R74 contacts a DNA residue, mostly the DNA right above and below the H3 tail exit site, but also up to 1 turn and more away from the exit point in this initial sampling phase (Supplementary Fig. S11e).

To ensure proper sampling of the conformational space also in the second stage of docking, we selected 1500 out of the best 3000 solutions according to their HADDOCK score for refinement in the (semi-) flexible phase (see also Supplementary Fig. S11d). Definition of interface residues in the PWWP-H3K36me3 complex was set to automatic. In addition residues 37-40 in the H3 tail were set to be fully flexible, all other atoms (including the DNA) were fixed. To favor solutions with small gaps across the cut-site of the H3-tail, a 1.2 Å unambiguous restraints for the peptide bond between residues 38 and 39 was enforced. To prevent that the H3 tail would be pulled out of the PWWP binding pocket, the interaction restraints from the first docking step were enforced as well.

The ligand rmsd over all solutions again shows that there are many favorable solutions with very different positions of the PWWP domain on the nucleosomal DNA. Residue R74 now shows a slightly more localized distribution of DNA contacts (Supplementary Fig. S11e). Note that at this point the gap between residues 38 and 39 varies between 3 and 20 Å.

In the final step, the best 750 solutions were refined in explicit water, and at the same time the covalent attachment of the two parts of the H3 tail was enforced by rebuilding it as a single chain. This allows the internal force field to recreate the peptide bond across 38 and 39 with the appropriate geometry. Residues 37-40 of H3 were set to be fully-flexible. In all structures, a proper

peptide bond was recreated. Overall, there is less spread in the solutions, although still a considerable range in ligand rmsd values, up to 35 Å (Supplementary Fig. S11d). Solutions without hydrogen bond (> 0.5 Å), dihedral angle (> 5°), or unambiguous restraint (> 0.5 Å) violations and with H3 tail (residues 37-40) backbone dihedrals in the core and allowed regions of the ramachandran plot (as defined by PROCHECK [10]) were ordered according to their HADDOCK score, the 10% highest (unfavourable) energy structures were removed, resulting in a final ensemble of 404 structures (filled squares in Supplementary Fig. S11d, left panel). As can be seen in Supplementary Fig. S11d, this procedure also filters out some solutions with favorable HADDOCK scores, which does not contain an energy term for ramachandran plot quality. In these structures, the peptide bond could only be recreated at expense of very bad backbone dihedrals for the linking residues.

The final ensemble of 404 solutions was first subjected to a clustering analysis, using the ligand backbone rmsd as defined above. Using a cut-off of 10 Å with a minimum of 10 cluster members, 91% of all structures were clustered into 7 clusters. The superposition of the 10 best structures for each cluster in Supplementary Figure S12a shows the distinct translational and rotational positions of the PWWP domain on the nucleosomal DNA.

Next, we looked at the residue-specific contributions to the intermolecular energies (sum of Vanderwaals and electrostatic terms) of all basic PWWP residues in the 50 best structures for the three largest clusters (Supplementary Table S1). Across all three clusters, active residues K16, K73, R74, and K75 make large contributions to the intermolecular energy, with K16 being overall most

important. Residues K67 and K70 were defined as active but contribute little to the intermolecular energy. Both residues indeed show a smaller effect in gel shift compared to others and have least amount of chemical shift perturbation.

Analysis of the contacts made by the other basic residues provides the opportunity to cross-validate the docking solutions, as we have gel-shift based mutation data for 4 of these residues, which were not included in the docking procedure. Cluster 3, 4, 6 and 7 are in conflict with the cross-validation data for both K39A and K56A mutations, while cluster 2 conflicts with the data for K56 and cluster 5 with that of K39. These clusters are therefore excluded from further consideration. The largest cluster of solutions, cluster 1, is agreement with all cross-validation data and has been deposited in the PDB (3ZH1).

Supplementary References

1. Vezzoli A, Bonadies N, Allen MD, Freund SMV, Santiveri CM, Kvinlaug BT, Huntly BJP, Göttgens B, Bycroft M: **Molecular basis of histone H3K36me3 recognition by the PWWP domain of Brpf1**. *Nat Struct Mol Biol* 2010, **17**:617–619.

2. Wu H, Zeng H, Lam R, Tempel W, Amaya MF, Xu C, Dombrovski L, Qiu W, Wang Y, Min J: **Structural and histone binding ability characterizations of human PWWP domains.** *PLoS ONE* 2011, **6**:e18919.

3. Luger K, Mäder AW, Richmond RK, Sargent DF, Richmond TJ: **Crystal structure of the nucleosome core particle at 2.8 A resolution**. *Nature* 1997, **389**:251–260.

4. Dominguez C, Boelens R, Bonvin AMJJ: **HADDOCK: a protein-protein docking approach based on biochemical or biophysical information**. *J Am Chem Soc* 2003, **125**:1731–1737.

5. Brünger AT, Adams PD, Clore GM, DeLano WL, Gros P, Grosse-Kunstleve RW, Jiang JS, Kuszewski J, Nilges M, Pannu NS, Read RJ, Rice LM, Simonson T, Warren GL: **Crystallography & NMR system: A new software suite for macromolecular structure determination**. *Acta Crystallogr D Biol Crystallogr*

1998, **54**:905–921.

6. Brunger AT: **Version 1.2 of the Crystallography and NMR system**. *Nature protocols* 2007, **2**:2728–2733.

7. Simon MD, Chu F, Racki LR, la Cruz de CC, Burlingame AL, Panning B, Narlikar GJ, Shokat KM: **The site-specific installation of methyl-lysine analogs into recombinant histones**. *Cell* 2007, **128**:1003–1012.

8. Karaca E, Bonvin AMJJ: **A multidomain flexible docking approach to deal with large conformational changes in the modeling of biomolecular complexes.** *Structure* 2011, **19**:555–565.

9. Clapier CR, Chakravarthy S, Petosa C, Fernández-Tornero C, Luger K, Muller CW: **Structure of the Drosophila nucleosome core particle highlights evolutionary constraints on the H2A-H2B histone dimer**. *Proteins* 2008, **71**:1–7.

10. Laskowski RAR, Rullmannn JAJ, MacArthur MWM, Kaptein RR, Thornton JMJ: **AQUA and PROCHECK-NMR: programs for checking the quality of protein structures solved by NMR.** *J Biomol NMR* 1996, **8**:477–486.
